# Longitudinal Analysis of Reproductive and Adrenal Hormones in Bontebok (*Damaliscus pygargus pygargus*)

**DOI:** 10.3390/ani16203171

**Published:** 2026-10-09

**Authors:** Candace D. Scarlata, Laurel B. F. Westcott, Michael Suhn, Nadja Wielebnowski

**Affiliations:** Oregon Zoo, 4001 SW Canyon Rd., Portland, OR 97221, USA; laurel.westcott@oregonzoo.org (L.B.F.W.); michael.suhn@oregonzoo.org (M.S.); nadja.wielebnowski@oregonzoo.org (N.W.)

**Keywords:** fecal hormone metabolites, reproductive physiology, adrenal activity, estrous cycle, gestation, seasonality, animal welfare, assay validation, bontebok, antelope

## Abstract

The bontebok (*Damaliscus pygargus pygargus*) is one of conservation’s most promising success stories, yet its reproductive and adrenal physiology has not been described, limiting evidence-based care of animals held under human management. We validated enzyme immunoassays for fecal progestagen, testosterone, and glucocorticoid metabolites and applied them to a longitudinal data set from six zoo-housed bontebok (three males, three females) sampled two to three times weekly over more than seven years. Assays were biochemically and biologically validated, with glucocorticoid metabolites rising markedly within 24 h of translocations and veterinary procedures. Progestagen monitoring characterized estrous cycles as 21 to 25 days and four pregnancies with a range of 223 to 236 days. Reproductive activity in this Northern Hemisphere population was displaced roughly six months relative to wild Southern Hemisphere populations. Males showed peak testosterone metabolites from June to October, and females cycled from September to January, implicating photoperiod as the possible driver of seasonality. Both sexes displayed seasonal glucocorticoid peaks in late spring and early summer despite constant nutrition, water, and social conditions. These findings provide the first endocrine reference base for the bontebok, are consistent with the theory that this subspecies is seasonally polyestrous with spontaneous ovulation, and support improved welfare monitoring and reproductive management.

## Simple Summary

The bontebok is a rare antelope from South Africa that was once nearly hunted to extinction but has since recovered through decades of careful conservation. Despite this success story, almost nothing has been published about the animal’s basic physiology, including the hormones that guide reproduction and the body’s response to stress. To help fill this gap, we studied a small group of bontebok by collecting fecal samples for more than seven years and analyzing them for reproductive and adrenal hormones. Using this approach, we tracked the females’ reproductive cycles and pregnancies, found seasonal changes in the males’ hormones relative to the breeding season, and measured increases in adrenal hormones in relation to specific events. We found that male testosterone peaked from June to October, coinciding with observed conceptions. Non-pregnant females cycled from September to January. This reproductive season is an approximately six-month shift relative to wild Southern Hemisphere populations. This work provides the first detailed physiological information for the bontebok, providing zoological institutions and conservation breeding centers with reference values to help guide breeding programs, monitor animal welfare, and help secure the long-term survival of this remarkable species.

## 1. Introduction

Bontebok (*Damaliscus pygargus pygargus)* are one of the rarest antelope species in Africa and a great example of how concerted conservation efforts helped bring a species back from the brink of extinction. This medium-sized antelope is endemic to the coastal grasslands of South Africa’s Western Cape and their dark brown coat is characterized by a striking white blaze on its nose and rump [1]. Bontebok, a member of the Bovidae family, exhibit a typical territorial bovid social system in which males establish and defend territories throughout the year and have a strong seasonal reproductive pattern [1,2]. In South Africa, bontebok mating season occurs from January to March and calving season occurs from September to November [2]. Bontebok look similar and are closely related to the blesbok (*Damaliscus pygargus phillipsi*), but they have been classified as a separate subspecies based on genetic differentiation, as well as behavioral and morphological differences [3]. Estimates suggest that bontebok started diverging from blesbok about 1.2 million years ago and achieved genetic separation about 12,800 years ago [4,5,6].

Historically, intensive hunting during the eighteenth and nineteenth centuries drove the wild bontebok population to near-extinction and by the early twentieth century, fewer than 20 individuals remained. The establishment of the Bontebok National Park in 1931, created explicitly to protect the remaining animals, enabled a remarkable population recovery to approximately 3500 individuals by 2008 [5]. This resurgence has been regarded as one of the most celebrated examples of conservation success in southern Africa; however, bontebok continue to be threatened by habitat fragmentation and low genetic diversity [4,5,7]. The conservation status of bontebok was changed from “vulnerable” to “least concern” by the IUCN in 2017 and they were removed from the CITES Appendix II in 2025 [7]. They are still listed as ‘Endangered’ under the United States of America’s Endangered Species Act; however, their status has not been re-evaluated since 1976. While current populations appear somewhat stable, there is concern about hybridization with blesbok and the loss of subspecies diversity due to limited control of animals in private ownership and on private lands [5].

Despite this long and well-documented conservation history, the basic physiology of bontebok remains poorly understood. Most critically, no published data exist on reproductive and/or adrenal hormone profiles for this subspecies. The characterization of such hormonal baselines is an important prerequisite, both for advancing fundamental knowledge of bontebok biology and for supporting evidence-based reproductive management as well as the welfare monitoring of individuals held under human care [8,9,10]. A better understanding of basic physiological responses and patterns can also be important for future conservation efforts. This study focused on the use of non-invasive fecal hormone monitoring, which allows for long-term hormonal tracking. Fecal-based methods help to eliminate the confounding effects of capture-induced stress and enable longitudinal data collection with minimal animal disturbance [11]. In addition, fecal hormone tracking has the advantage of providing a pooled estimate of hormone production from the previous hours to days, depending on the metabolic excretion rate of the species, rather than a “point in time” sample obtained in serum [12]. This helps to minimize acute and diurnal fluctuations and thus allows us to get a better picture of longer-term hormonal changes [10]. When properly validated, fecal hormone metabolite analyses represent a powerful and practical framework for addressing fundamental questions in reproductive physiology, stress biology, animal welfare, and conservation management across a broad range of taxa [8,9,10,11,12,13,14,15,16,17].

In 2015, the Oregon Zoo (Portland, OR, USA) received a pair of male bontebok as part of broader institutional efforts to develop and maintain an ex situ population of this species. Shortly after their arrival, excessive sparring between the two males necessitated management intervention: one male was relocated to another institution, and a female was subsequently acquired to form a stable breeding pair with the remaining male. The male and female had successful breeding events and two bontebok offspring were added to the overall population. As part of the Oregon Zoo’s ongoing animal welfare monitoring program, fecal samples for hormone metabolite analyses were collected, generating a longitudinal data set spanning more than 7 years. To the best of our knowledge, the present study therefore represents the most extensive body of data on reproductive and adrenal hormone fluctuations for this species to date.

The specific objectives of this study were to:

(1) Validate fecal adrenal and reproductive hormone metabolite enzyme immunoassays (EIAs) for male and female bontebok;
(2) Characterize the reproductive biology of female bontebok by monitoring progestagen concentrations during estrous cycles, pregnancy and contraception;
(3) Characterize hormonal changes in males associated with reproduction and season;
(4) Monitor variations in adrenal hormones under various conditions and seasons.

The data generated will contribute to the emerging body of conservation physiology knowledge for African antelope. It is expected to provide an important endocrine reference base for reproductive management, animal care and welfare assessments of bontebok, and ultimately may help to support the broader goal of sustaining this remarkable species for future generations.

## 2. Materials and Methods

### 2.1. Study Animals and Fecal Collections

For this study, fecal samples were collected 2–3 times a week in the morning (7 am–11 am) from 6 bontebok (3.3). Samples were frozen immediately and stored in −20° Celcius freezers until analysis. All study animals were captive-born in the Northern Hemisphere (Table 1). During the study, all animals were housed at the Oregon Zoo (Portland, OR, USA, latitude: 45′ N) with the exception of (1) May (#460), who was housed at the Binder Park Zoo (Battle Creek, MI, USA, latitude: 42′ N), and (2) Winter (#479), who was housed at the Oregon Zoo during most of the study, but was transferred to the Binder Park Zoo on 7 October 2025.

Karoo (#471) and Bhisho (#472) were transferred to the Oregon Zoo in July 2015, but excessive sparring between the two males led to the transfer of Bhisho to another facility in September 2016. Winter (#479) was transferred to the Oregon Zoo on 8 June 2018, and was reproductively active for the next 5 years. In the sixth year, Winter was placed on oral contraception (Melengestrol acetate (1.15 mg/mL), 0.5 mg once a day (sid) for 90 days, Wedgewood, Swedesboro, NJ, USA) in October 2023 to prevent further breeding. In the seventh year, she was taken off contraception in May 2025 and transferred to the Binder Park Zoo on 7 October 2025. When Winter was transferred to the Binder Park Zoo, this facility continued collecting fecal samples.

At this time, we also took the opportunity to add May (#460), another female housed at the Binder Park Zoo, to our study. This female was housed at the Binder Park Zoo for 7 years and bred successfully 5 times before she was included in this study. Tutula (#556, 0.1) was the 1st surviving offspring of Winter and Karoo. She was orally contracepted (Melengestrol acetate (1.15 mg/mL), 0.5 mg [0.44 mL] once a day (sid) for 90 days, Wedgewood) in March 2023 and was transferred to another facility in May 2024. Monte (#590, 1.0), the 2nd surviving offspring of Winter and Karoo, was housed at the Oregon Zoo until he was 8 months old, when he was transferred to another facility.

### 2.2. Steroid Extraction

Steroids were extracted using an adaptation of methods documented by Fink et al., 2021 [18]. Briefly, 0.500 g (±0.025 g) of wet, homogenized fecal matter was mixed with 5 mL 80% ethanol, vortexed, and shaken overnight for 17 h (Fisherbrand^TM^ open air rocking shaker, Pittsburgh, PA, USA). Then, the samples were vortexed again and centrifuged (Sorvall ST^TM^16, Waltham, MA, USA) at 2500 rpm for 15 min. Three milliliters of the supernatant were saved, and dilutions were then created with assay buffer based on optimal concentrations for the detection of different steroids. If the optimal dilution was less than 1:16, 500 μL of the neat extract was dried completely in a speedvac (Savant^TM^ speedvac DNA130, Waltham, MA, USA) and reconstituted in 500 μL of Tris assay buffer before being diluted to remove any possible effect of the ethanol on assay results.

### 2.3. Enzyme Immunoassay (EIA) Analysis

Fecal progestagen metabolites (FPMs) were analyzed using a double-antibody progesterone enzyme immunoassay (EIA). The EIA used a monoclonal antibody produced against 4-pregnen-11-ol-3,20-dione Hemisuccinate:BSA (CL425, 1:100,000; C. Munro, University of California, Davis, CA, USA), a horseradish peroxidase-conjugated progesterone label (1:50,000) and progesterone standards (0.024–6.25 ng/mL, Sigma Chemical Co., St. Louis, MO, USA). Microtiter plates were pre-coated with secondary goat anti-mouse IgG antibody (150 μL/well at 0.10 mg/mL, A008, Arbor Assays, Ann Arbor, MI, USA) using a process developed by Arbor Assays. The sensitivity of the assay was 0.033 ng/mL. The inter- and intra-assay coefficients of variation (CVs) were maintained below 15% and 10%, respectively, for this assay. Progesterone antibody cross-reactivities were 100% with progesterone, 172% with 3β-hydroxy-progesterone, 188% with 3α-hydroxyprogesterone, 2.7% with 11β-hydroxy-progesterone, 147% with 11α-hydroxy-progesterone, 7.0% with 5α-dihydroprogesterone, 5.9% with pregnenolone and less than 0.10% with corticosterone and androstenedione (C. Munro, University of California, Davis, CA, USA).

Fecal testosterone metabolites (FTMs) were analyzed using a double-antibody testosterone EIA previously described in Glaeser et al., 2022 [19]. The EIA used a polyclonal antibody produced against testosterone (R156/7, 1:187,500; C. Munro, University of California, Davis, CA, USA), a horseradish peroxidase-conjugated testosterone label (1:100,000) and testosterone standards (0.023–6.0 ng/mL). The sensitivity of the assay was 0.015 ng/mL. The inter- and intra-assay CVs were maintained below 15% and 10%, respectively, for this assay. Testosterone antibody cross-reactivities were 100% with testosterone, 57.37% with 5α-dihydrotestosterone, 0.27% with androstenedione and less than 0.10% for 17 other steroids tested (C. Munro, University of California, Davis, CA, USA).

Fecal glucocorticoid metabolites (FGMs) were analyzed using a double-antibody corticosterone EIA previously described in Fink et al., 2021 [18]. The EIA used a polyclonal antibody produced against corticosterone-3-CMO-BSA (CJM006, 1:100,000; C. Munro, University of California, Davis, CA, USA), a horseradish peroxidase-conjugated corticosterone label (1:100,000) and corticosterone standards (0.078–20.0 ng/mL, C151, Arbor Assays, Ann Arbor, MI, USA). The sensitivity of the assay was 0.100 ng/mL. The inter- and intra-assay CVs were maintained below 15% and 10%, respectively, for this assay. Corticosterone antibody cross-reactivities were 100% with corticosterone, 14.25% with desoxycorticosterone, 2.65% with progesterone, 0.90% with tetrahydrocorticosterone, 0.64% with testosterone, 0.23% with cortisol, and less than 0.10% for 5 other steroids tested (C. Munro, University of California, Davis, CA, USA). For both the testosterone and corticosterone EIAs, microtiter plates were pre-coated with secondary goat anti-rabbit IgG antibody (150 μL/well at 0.10 mg/mL, A009, Arbor Assays, Ann Arbor, MI, USA) using a process developed by Arbor Assays.

### 2.4. Biochemical and Biological Validation

**Figure 1:**
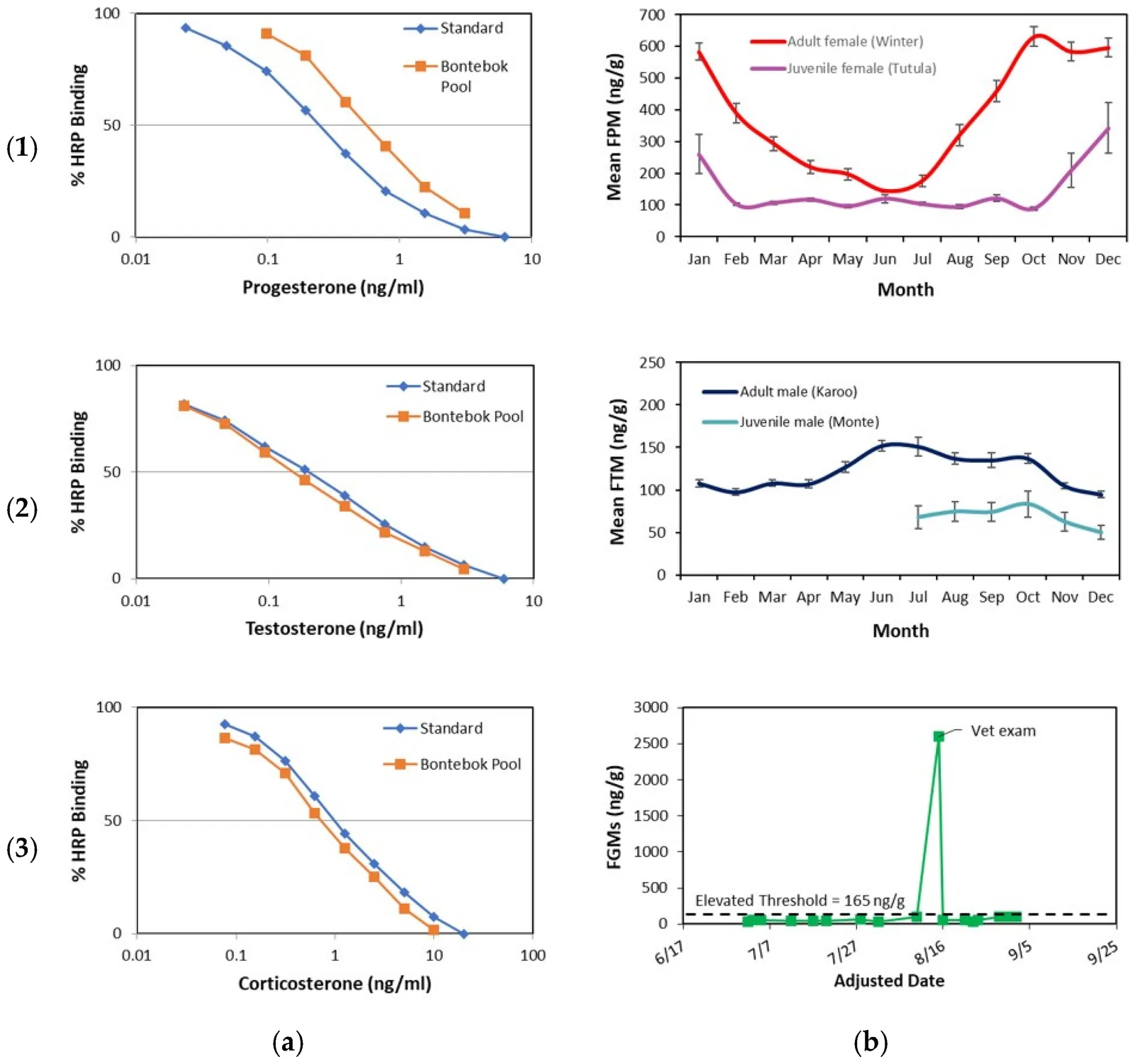
Endocrinology validation data for bontebok assays: (**1**) progesterone, (**2**) testosterone, (**3**) corticosterone. Column (**a**) shows the parallelism of pooled samples and hormone standards. Column (**b**) shows biological validations. Adjusted date = the date of sample collection minus one day. FPMs = fecal progestagen metabolites; FTMs = fecal testosterone metabolites; FGMs = fecal glucocorticoid metabolites.

Each EIA was biochemically validated by demonstrating: (1) parallelism between binding inhibition curves of dilutions of bontebok fecal extracts and the respective standard curve, and (2) significant recovery of exogenously added steroid (>90%) to fecal extracts before analysis (Figure 1a). Biological validation for progestagen and testosterone was determined by observing significant differences in hormone concentrations between different reproductive stages, such as between adult and juvenile animals of the same sex (Figure 1b), as well as significant differences within an animal during different reproductive stages. Biological validation for glucocorticoids in each sex was determined by observing a significant increase in FGMs over baseline approximately 24 h after a stressful event such as the transfer of the animal between facilities or after a veterinary exam (Figure 1b).

Recovery Results:

FPM Recovery: 94.1%, y = 0.959 x + 0.003, R^2^ = 0.998.

FTM Recovery: 97.7%, y = 0.989 x − 0.004, R^2^ = 0.998.

FGM Recovery: 91.1%, y = 0.901 x + 0.004, R^2^ = 0.998.

### 2.5. Data Analysis

For each individual, a baseline mean was calculated for each steroid using an iterative process in which high values exceeding the mean + 2 standard deviations (SDs) were removed until all extreme values were excluded. Baseline mean plus 2 SDs was used as a “threshold” for identifying elevated values for testosterone and glucocorticoid values. For glucocorticoids, values above the threshold were common after a stressful event. For the physiological validation of FGM, we reported the percent difference between FGM concentrations in samples associated with a stressful event and the calculated FGM baseline.

To identify elevated progestagen values during luteal phases and pregnancy, the overall mean plus 1.5 SDs from samples collected during the reproductive season was used as a threshold. Not enough samples were collected from Tutula and May for a proper calculation, so Winter’s threshold was used to identify elevated luteal phase samples in these females. Estrous cycle length was calculated as the number of days between the beginning of one luteal phase and the beginning of the next luteal phase, which was based on a sustained elevation of FPM concentrations above the elevated threshold for at least 3 consecutive samples. Gestation length (or length of elevated FPM concentrations during gestation) was calculated as the number of days between the last baseline FPM sample and the date of parturition. In longitudinal graphs, we use “Adjusted Date” to represent the date of sample collection minus one day. This accounts for the lag time between the secretion and excretion of hormones. This was based on the lag time we observed (~24 h, Figure 1b) and lag times reported in other ungulate species. For example, in the closely related blue wildebeest (*Connochaetes taurinus*), an ACTH challenge showed a significant increase in glucocorticoids above baseline 23 h post-injection for a male and female [20,21]. In impala (*Aepyceros melanmpus*), peak glucocorticoids were detected 20 h post-ACTH injection [22]. And in the sable antelope (*Hippotragus niger*), the lag time from the injection of progesterone to peak fecal excretion was 16 h [23].

All data analyses were performed using the R statistical package (version 4.4.3). Data are presented as mean ± standard error of the mean (SE) and sample size (*n*). A Shapiro–Wilk test of normality showed that all 3 hormone data sets were non-normal, so each hormone data set was log-transformed to account for non-normality. A median was also reported for steroid hormone values due to non-normality. Statistical significance was set to *p* < 0.05. A Pearson’s product-moment correlation was used to determine the relationship between hormone values obtained from the same sample (FPM vs. FGM and FTM vs. FGM). For the physiological validation of FPM, a repeated-measures ANOVA was used to look at the relationship among log (FPM) in all 3 females and 5 reproductive stages: juvenile, estrous cycling, pregnancy, contraception and non-contracepted adults during the non-breeding season. For the physiological validation of FTM, a repeated-measures ANOVA was used to look at the relationship between adult and juvenile FTM concentrations in all 3 males. For the comparison of hormone concentrations within and between pregnancies, a repeated-measures ANOVA was used to look at the relationship of log (FPM) or log (FGM) to both pregnancy number (Pregnancy 1, 2, 3 or 4) and gestational period (early = Days 1–75, middle = Days 76–150, late = Days 151–parturition).

Seasonal analyses for each hormone were conducted using a repeated measures ANOVA test between log (hormone) and several factors: (1) seasonality (as estimated by month), (2) age (rounded to the nearest whole number), (3) animal and (4) reproductive stage (for females only). ‘Animal’ was aliased with age and thus was not a significant factor in any of the seasonal analyses. If a factor was significant in the model, then Tukey’s HSD test was used to determine which groups within a factor were significantly different. We also removed any elevated FGM data points related to stressful human interventions, like veterinary exams or transfers, that might obscure seasonal effects. Animal ‘May’ was excluded from the seasonal analyses because we only had 3 months of cycling data from her. Karoo was the only male with more than 1 year of data, so for the seasonal analyses in males, we conducted a full model with all 3 males and a smaller model with only Karoo’s data (1-male model).

## 3. Results

### 3.1. Fecal Hormone Metabolite (FPM and FGM) Patterns in Females

#### 3.1.1. Biological Validation of FPMs and FGMs for Females

Biological validation of the progestagen assay was confirmed by significant differences in FPM concentrations and patterns when compared between five reproductive stages: (1) juvenile, (2) non-breeding season in adults, (3) estrous cycles, (4) pregnancy, and (5) contraception. An ANOVA found significant differences between log FPM concentrations and all five reproductive stages (F (4, 1472) = 1879, *p* < 0.001, *η*^2^ = 0.836), with the exception of juvenile and contracepted females (Table 2, Figure 1(1b)). FPM concentrations in the juvenile female averaged 123 ± 12.3 ng/g (*n* = 42) in comparison to 158 ± 4.00 ng/g (*n* = 523) in the adult, intact female during the non-breeding season.

**Figure 2:**
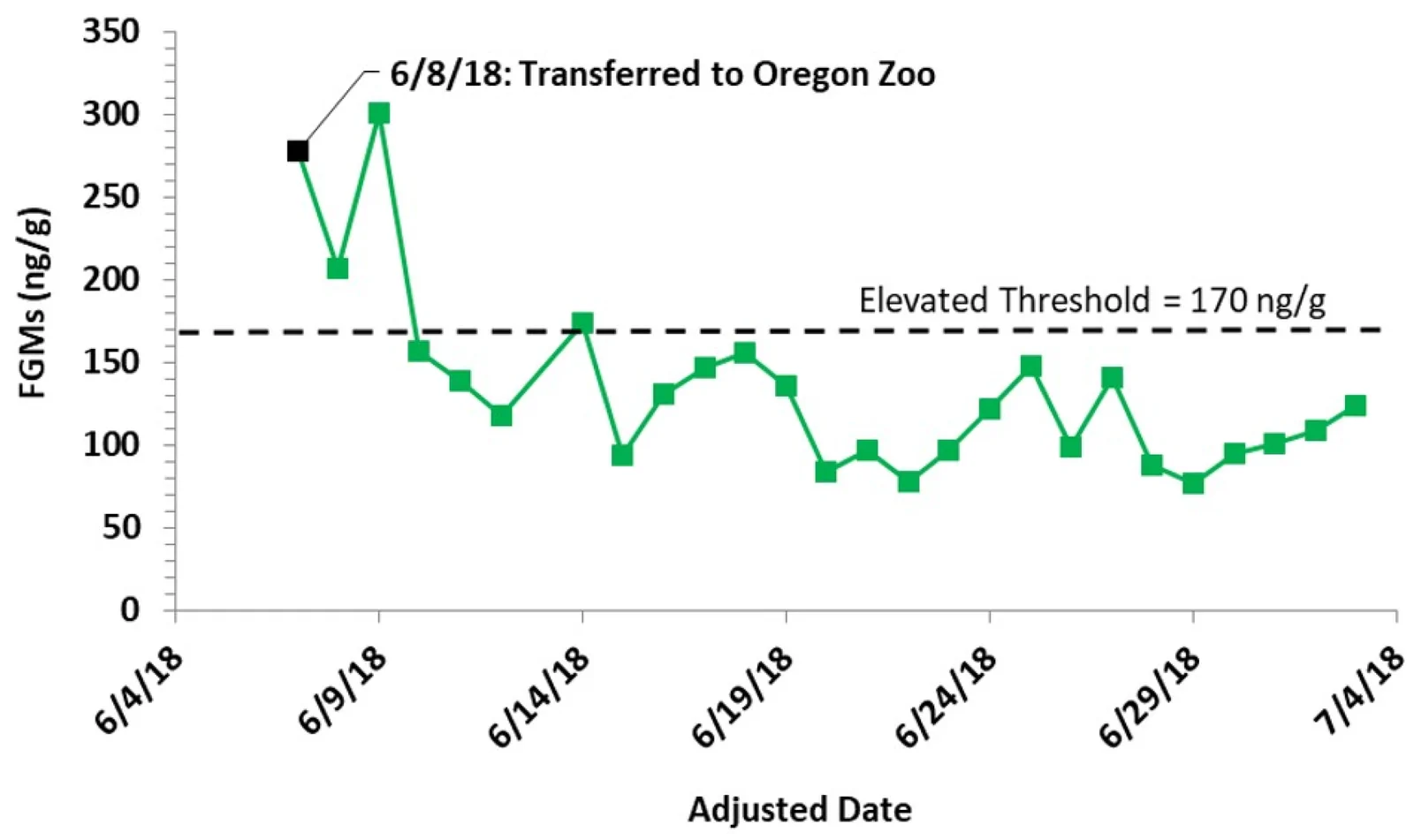
FGM patterns in an adult female bontebok (Winter) after transfer to the Oregon Zoo. Dotted line = elevated FGM threshold. Values above this line are considered “elevated.” Black dot indicates the FGM value associated with the day of her inter-facility transfer. Adjusted date = the date of sample collection minus one day.

Biological validation of the glucocorticoid assay was confirmed in our female Winter (#479) during an inter-facility transfer event to the Oregon Zoo. We observed substantially elevated FGM concentrations within 24 h of her arrival at the Oregon Zoo, and after 3 days, her FGM concentrations declined to baseline, indicating acclimation to her new surroundings (Figure 2). FGM concentrations during her first 3 days at the Oregon Zoo averaged 262 ng/g, which is a 300% increase over baseline (baseline FGM = 87.8 ng/g; elevated threshold = 170 ng/g).

#### 3.1.2. Characteristics of the Estrous Cycle

Fifteen complete estrous cycles in three different females were observed. Winter (#479) had estrous cycles from September 2018 to January 2019. For the next 4 years, she conceived annually between June and September, before any estrous cycles were observed. In the sixth year, Winter showed evidence of estrous cycling in September 2023 but was placed on oral contraception in October 2023 to prevent further breeding. In the seventh year, Winter was taken off contraception in May 2025 and she cycled from September 2025 to January 2026. May (#460) showed estrous cycles from October 2025 to January 2026. However, fecal collections for her did not start until October and we may have missed the beginning of the cycling season for her. Tutula (#556) showed her first signs of progestagen cycling in November 2022 when she was 9 months old, and she continued to cycle until January 2023, before she was orally contracepted in March 2023.

**Figure 3:**
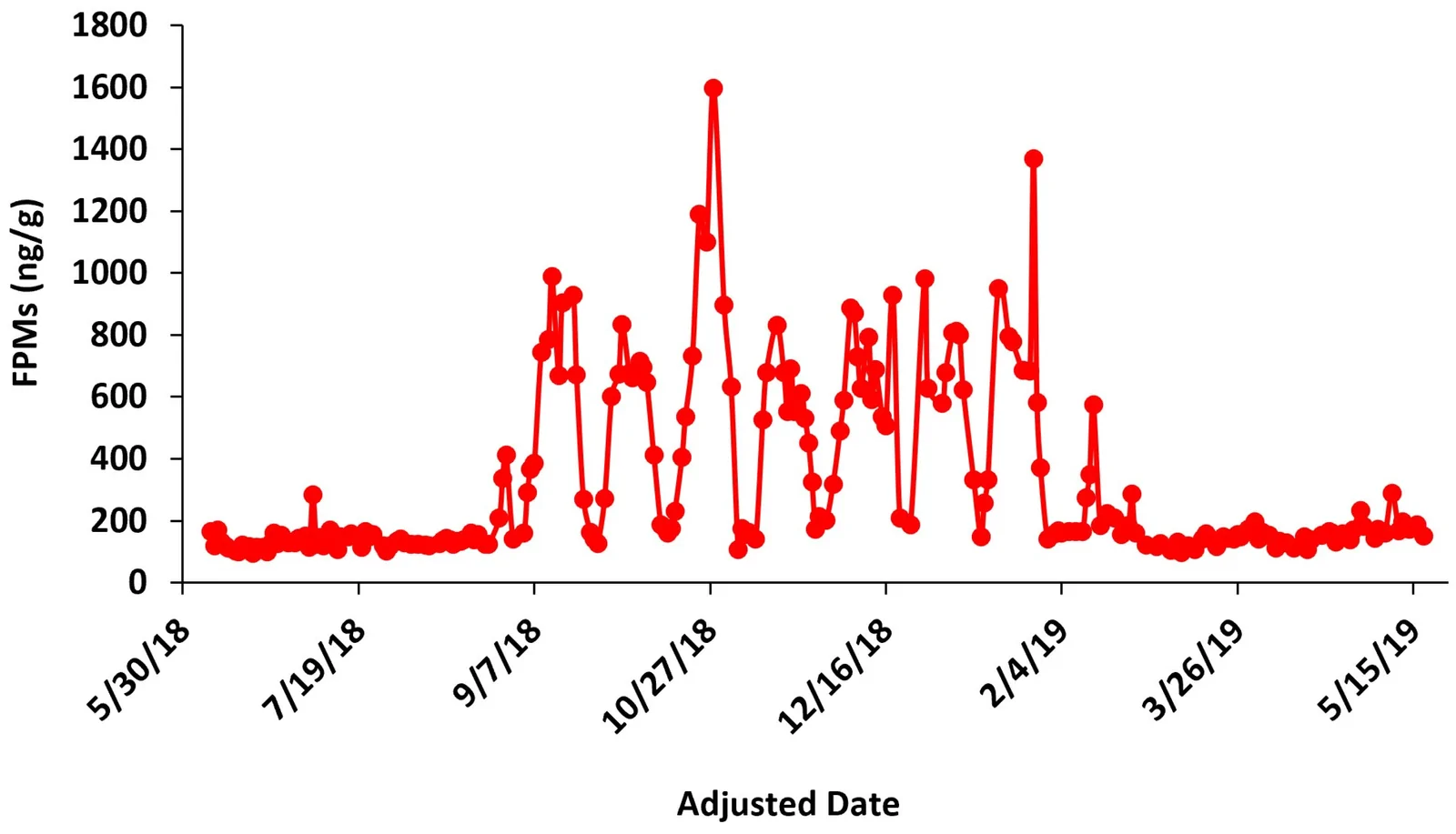
Fecal progestagen metabolite (FPM) patterns during 7 estrous cycles in an adult female bontebok (Winter). Adjusted date = the date of sample collection minus one day.

Estrous cycle length had a mean duration of 22.1 ± 0.29 days (range: 21–25 days, *n* = 15, Figure 3). The luteal phase, as defined by elevated FPM values, lasted 14.7 ± 0.20 days (range: 13–16 days, *n* = 19) and the inter-luteal phase lasted 7.27 ± 0.23 days (range: 6–9 days, *n* = 15). FPM concentrations during the luteal phase averaged 666 ± 211 ng/g (range: 322–1100 ng/g, *n* = 131; see Table 3 for each female’s individual means and medians).

#### 3.1.3. Characteristics of Pregnancy

Four pregnancies in one female (Winter, #479) were monitored over the course of our study. The calf in her first pregnancy was stillborn and the calf in the second pregnancy died after only 28 days. The 3rd and 4th pregnancies were successful and produced 2 of our study animals, Tutula (#556) and Monte (#590).

The mean length of FPM elevation during gestation was 231 days (range = 223–236 days, SE = 2.90, *n* = 4 pregnancies). This value approximates the minimum gestation length of bontebok, but actual gestation may be longer because the exact date of breeding was not observed, and FPM concentrations may take some time to increase after conception. Mean FPM concentrations during gestation were 739 ± 8.90 ng/g (range: 91.0–1542 ng/g, *n* = 401). There was significant overlap between luteal phase and gestational FPM concentrations. Therefore, pregnancy cannot be distinguished from luteal phases by a single sample test but requires longitudinal monitoring. Interbirth intervals ranged from 304 to 425 days (Table 4).

**Figure 4:**
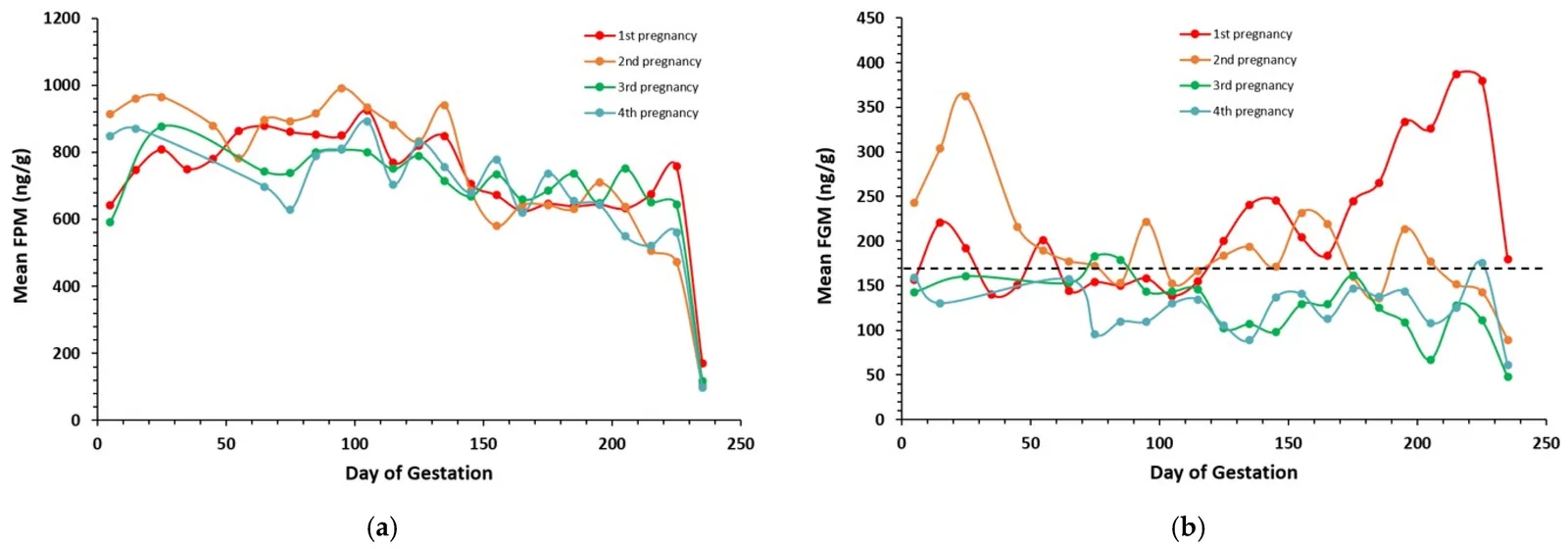
(**a**) FPM and (**b**) FGM means over 10-day periods during 4 pregnancies in 1 female bontebok. Dotted line = elevated FGM threshold.

Also, we saw an interesting FPM pattern in all four pregnancies that suggests that FPM concentrations may decline slightly during late gestation (Table 4, Figure 4). An ANOVA found significant differences between log FPM concentrations during early, middle and late gestation (gestational period: F (2, 396) = 40.2, *p* < 0.001, *η*^2^ = 0.172), with late gestation having significantly lower FPM concentrations than early and middle gestation. There were no FPM differences between pregnancies (pregnancy number: F (3, 396) = 1.03, *p* = 0.379, *η*^2^ = 0.006). Because Winter’s first pregnancy resulted in a stillborn calf, zoo veterinary staff were concerned that this dip in FPMs at the end of pregnancy (Figure 4) may have affected her ability to sustain the pregnancy properly. Therefore, during the next 3 pregnancies, the veterinarians decided to administer Regu-Mate^®^ (2.2 mg altrenogest per mL (0.22%), Intervet/Merck Animal Health, Oral Solution for Horses, Rahway, NJ, USA), a medication used to support pregnancy maintenance in some other species, around day 180 of pregnancy [24]. All three subsequent pregnancies resulted in the birth of a live calf; however, despite the use of Regu-Mate^®^, we continued to see a similar decrease in FPM concentrations during late gestation, and thus, it is unlikely that this decline affected pregnancy maintenance. Lastly, samples collected on the day of parturition showed FPM concentrations lower than the elevated threshold of 345 ng/g (FPM mean of samples collected the day of parturition = 316 ng/g, *n* = 3 pregnancies). If we take into account the approximate 1-day time lag between secretion and excretion of this hormone, this suggests FPM concentrations probably started to drop at least 1 day before parturition.

Glucocorticoids also play an important role during pregnancy and parturition [17,25]. We therefore analyzed FGM concentrations during early (Days 1–75), middle (Days 76–150) and late (Days 151–parturition) gestation and saw some interesting patterns when comparing FGM means among the four pregnancies. An ANOVA found significant differences in FGMs between pregnancies (pregnancy number: F (3, 396) = 49.3, *p* < 0.001, *η*^2^ = 0.263), with pregnancies 1 and 2 having higher FGM concentrations than pregnancies 3 and 4. Notably, FGM means for early, middle and late gestation in the first 2 pregnancies were all above the elevated threshold (170 ng/g), while FGM means for early, middle and late gestation for the last 2 pregnancies were all below the elevated threshold (Figure 4, Table 4). In addition, we found significant differences between FGM concentrations during early, middle and late gestation (gestational period: F (2, 396) = 4.75, *p* = 0.009, *η*^2^ = 0.032), with middle gestation having significantly lower FGM concentrations than early and late gestation.

#### 3.1.4. Seasonal Changes in FPMs and FGMs for Females

**Figure 5:**
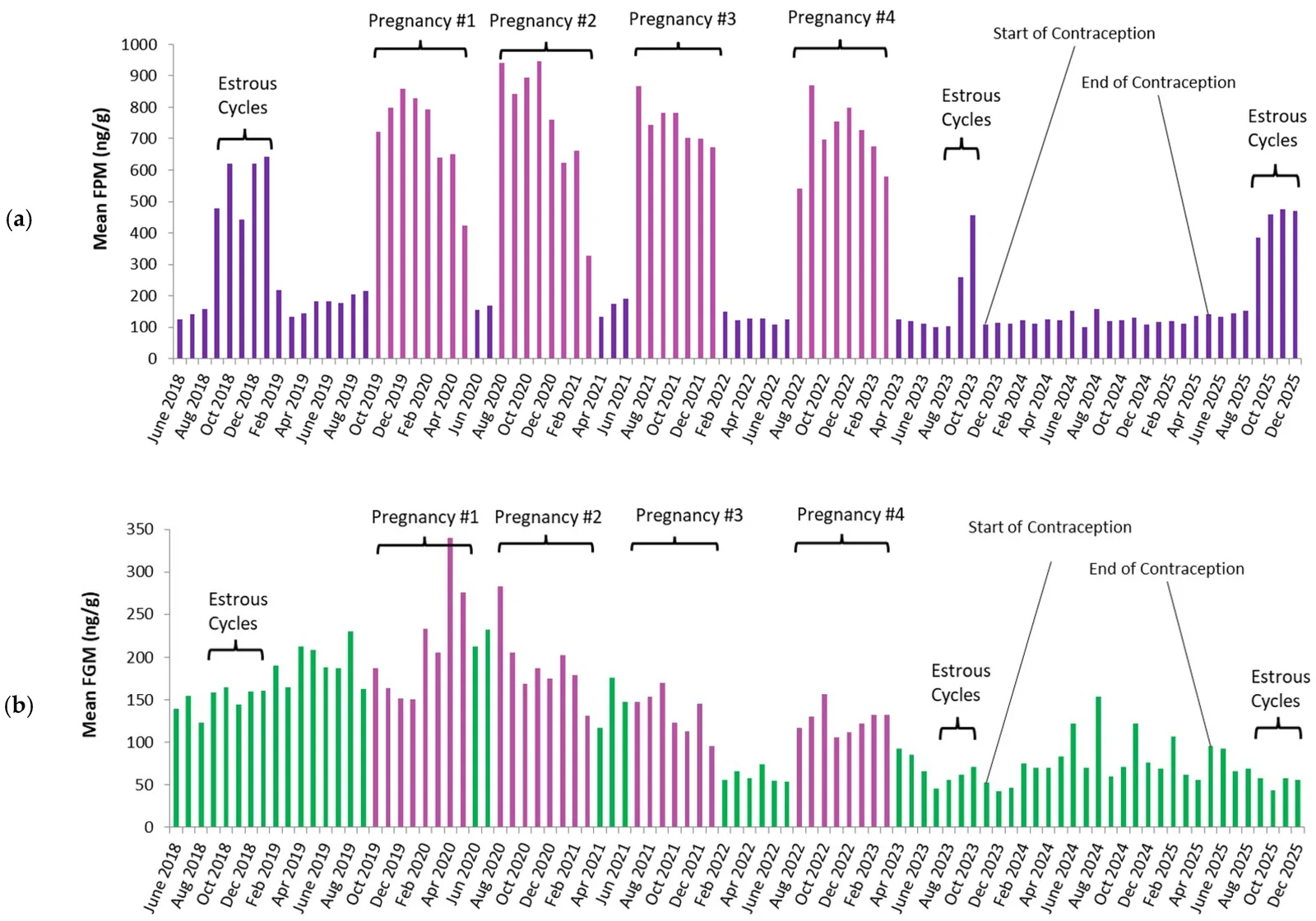
Longitudinal patterns in monthly (**a**) FPM and (**b**) FGM means in an adult female bontebok (Winter) showing changes during estrous cycling, pregnancy, and contraception over 7 years. Pink bars are meant to highlight months where the female was pregnant for the majority of the samples collected.

**Figure 6:**
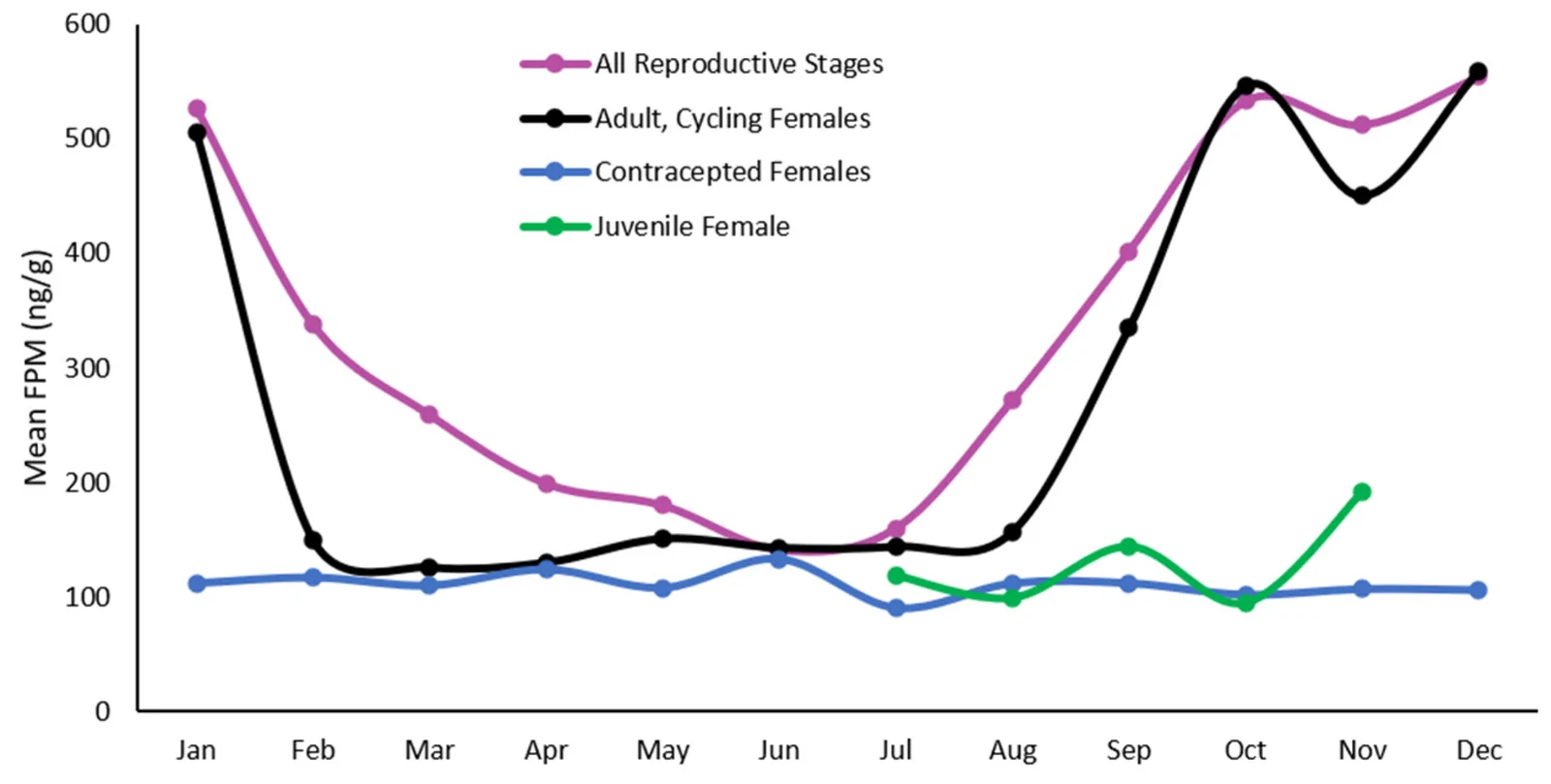
FPM means by month for 3 different reproductive stages compared to the overall mean of all reproductive stages in 3 female bontebok.

**Figure 7:**
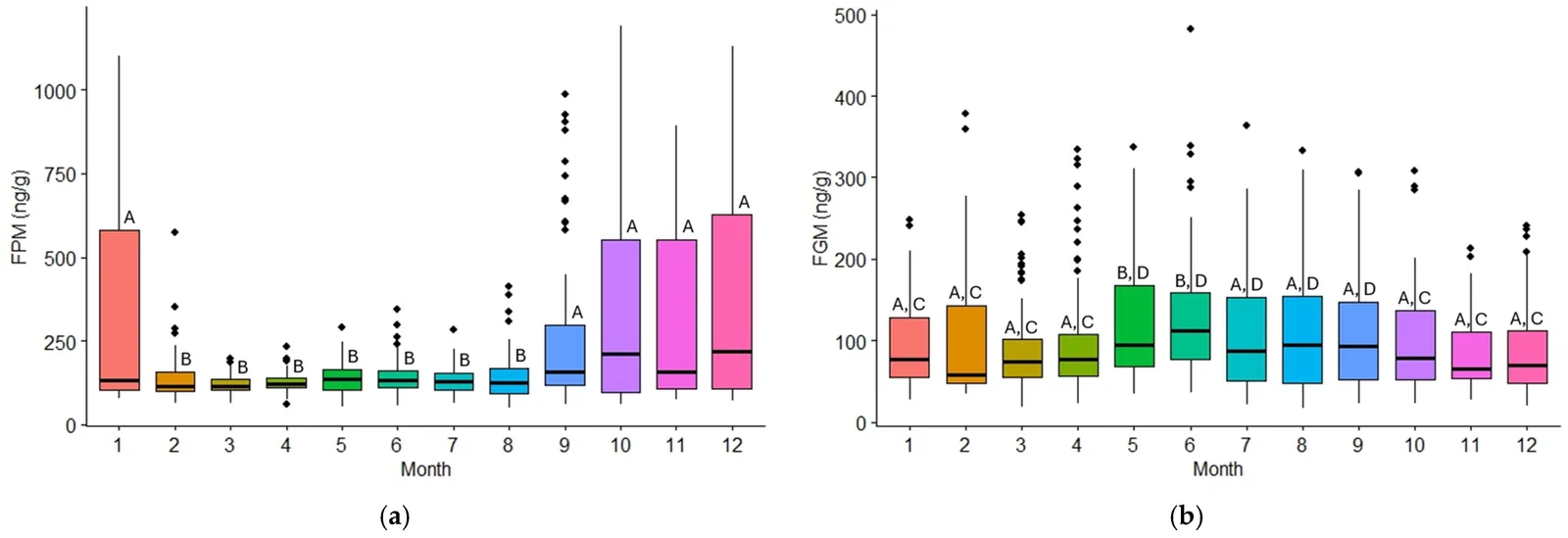
Seasonal changes in monthly (**a**) FPMs and (**b**) FGMs means for 3 female bontebok (excluding pregnancy samples). Months with different letters (A, B, C or D) are significantly different from one another (*p* < 0.05, Tukey’s HSD test). Box plots show the median (center line), interquartile range (box), 1.5× interquartile range (whiskers), and individual outliers (rhombus).

As expected, FPM concentrations were higher during the reproductive season due to increased progestagen secretion during estrous cycling and pregnancy, but this difference disappeared when females were contracepted (Figure 5 and Figure 6). When analyzing seasonal changes in FPMs, the full ANOVA model showed that month, age and reproductive stage were all significant factors (month: F (11, 1424) = 3.11, *p* < 0.001, *η*^2^ = 0.241, age: F (12, 1424) = 13.1, *p* < 0.001, *η*^2^ = 0.368, reproductive stage: F (4, 1424) = 661, *p* < 0.001, *η*^2^ = 0.254). Based on Tukey’s HSD, there were significant differences between many of the reproductive stages (Figure 5 and Figure 6, Supplemental Table S1), so for the final model, only samples from non-pregnant females were used (Figure 7a). For this model, month, age and reproductive stage were significant (month: F (11, 1023) = 2.17, *p* = 0.014, *η*^2^ = 0.178, ge: F (11, 1023) = 12.7, *p* < 0.001, *η*^2^ = 0.264, reproductive stage: F (3, 1023) = 261, *p* < 0.001, *η*^2^ = 0.242). FPM concentrations from February to August were significantly lower than FPM concentrations from September to January (Figure 6 and Figure 7a). September through January were also the same months during which we observed estrous cycles. However, our pregnancy data suggest that the female fertility window may start as early as June.

When analyzing seasonal changes in FGMs for females, the full ANOVA model showed that month, age and reproductive stage were all significant factors (month: F (11, 1424) = 8.68, *p* < 0.001, *η*^2^ = 0.009, age: F (11, 1424) = 88.7, *p* < 0.001, *η*^2^ = 0.545, reproductive stage: F (4, 1424) = 55.6, *p* < 0.001, *η*^2^ = 0.060). Based on Tukey’s HSD, pregnancy-related data were significantly higher than any other reproductive stage. We therefore removed pregnancy data for the final analysis. In the final model, month and age were significant, but reproductive stage was not (month: F (11, 1023) = 4.01, *p* < 0.001, *η*^2^ = 0.046; age: F (11, 1023) = 82.5, *p* < 0.001, *η*^2^ = 0.529, reproductive stage: F (3, 1023) = 2.75, *p* = 0.052, *η*^2^ = 0.003).

Results showed that FGM concentrations (with pregnancy samples removed) were the highest in May and June, followed by July, August and September (Figure 7b). In addition, we saw significantly higher FGM concentrations at ages 3–6 than at ages 7–11. For Winter specifically, ages 3–6 correspond to the years she cycled, was introduced to the male and had her first and second pregnancies, while ages 7–11 correspond to the years of her third and fourth pregnancy and subsequent contraception. Although there was a significant, yet moderate, positive correlation between FPMs and FGMs within samples (r (1475) = 0.577, *p* < 0.001), we did observe independent secretion patterns in these two hormones (Figure 5 and Figure 7).

### 3.2. Fecal Hormone Metabolite (FTM and FGM) Patterns in Males

#### 3.2.1. Biological Validation of FTMs and FGMs in Males

**Figure 8:**
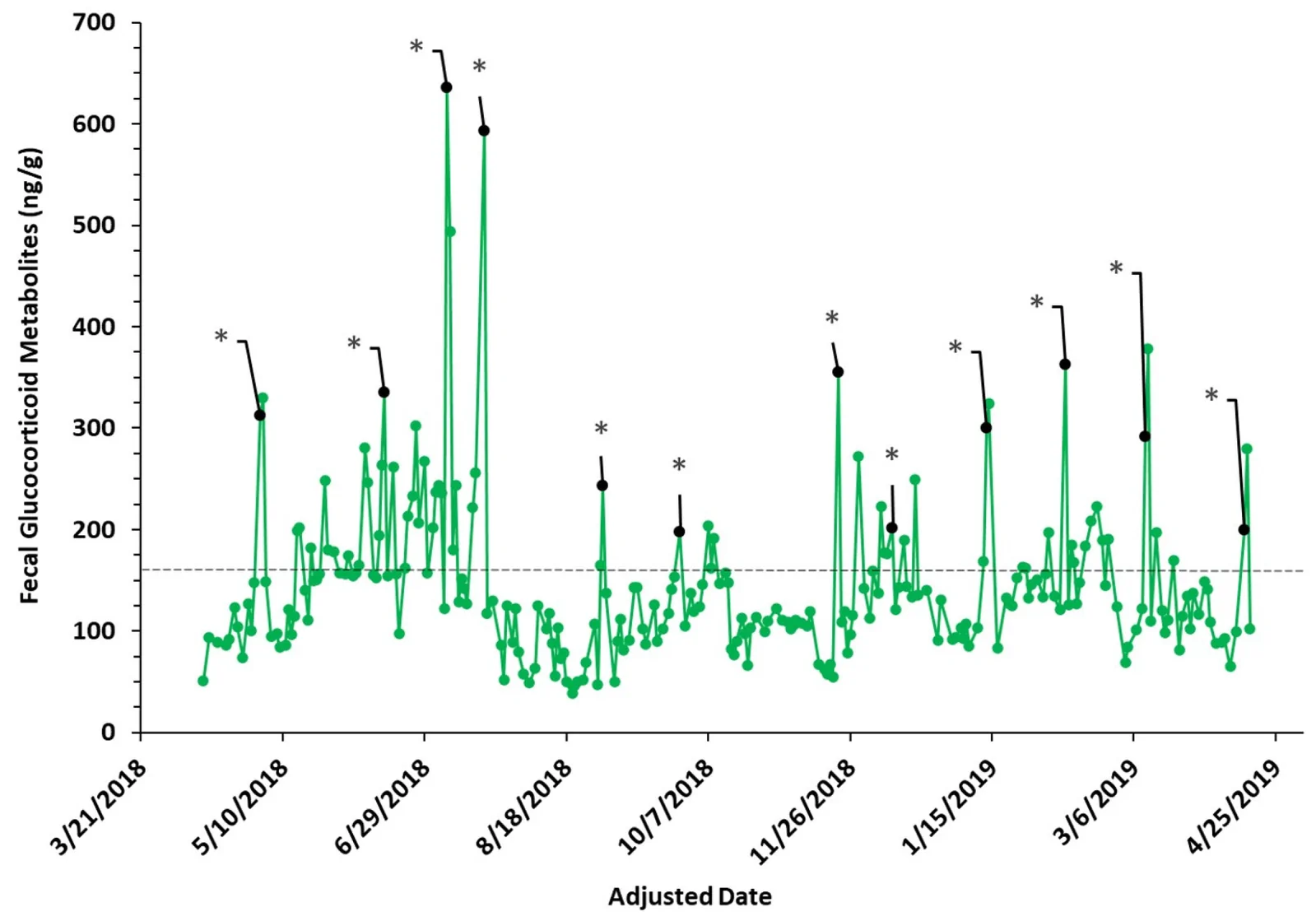
FGM patterns in an adult male bontebok (Karoo) over a 1-year period. Dotted line = elevated FGM threshold. Values above the dotted line are considered “elevated.” Asterisks (*) pointing to black dots = dates the male was anesthetized for veterinary exams. Adjusted date = the date of sample collection minus one day.

Adult male bontebok showed significantly higher FTM concentrations than a juvenile male bontebok (F (11,228) = 113, *p* < 0.001, *η*^2^ = 0.085), suggesting a biological validation of the testosterone assay (Figure 1(2b), Table 5). Biological validation of the glucocorticoid assay was confirmed for males by observing a significant increase in FGM concentrations in samples collected the day after several veterinary exams. In 2018, our male bontebok Karoo (#471) presented with hoof overgrowth and abnormal hoof conformation that required him to be anesthetized almost monthly for corrective hoof trimming and application of a corrective apparatus. Karoo showed significantly elevated FGMs in samples collected the day after these 12 veterinary exams (Figure 8), suggesting a biological validation of the glucocorticoid assay. His mean FGM concentration one day after veterinary exams was 336 ng/g, which is a 364% increase over baseline (baseline FGM = 92.1 ng/g, elevated threshold = 165 ng/g, range = 198–636, SE = 41.3, *n* = 12).

#### 3.2.2. Seasonal Changes in FTMs and FGMs for Males

**Figure 9:**
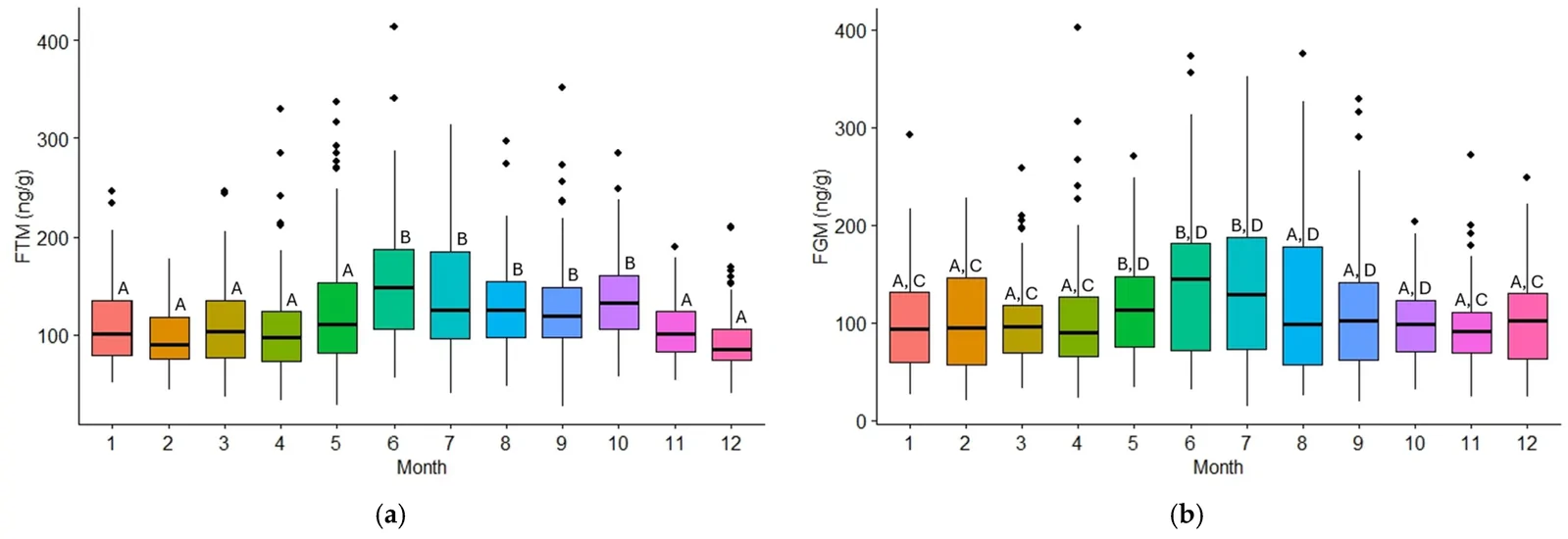
Seasonal changes in (**a**) FTM and (**b**) FGM concentrations in 1 adult male bontebok (Karoo). Months with different letters (A, B, C or D) are significantly different from one another (*p* < 0.05, Tukey’s HSD test). Box plots show the median (center line), interquartile range (box), 1.5× interquartile range (whiskers), and individual outliers (rhombus).

**Figure 10:**
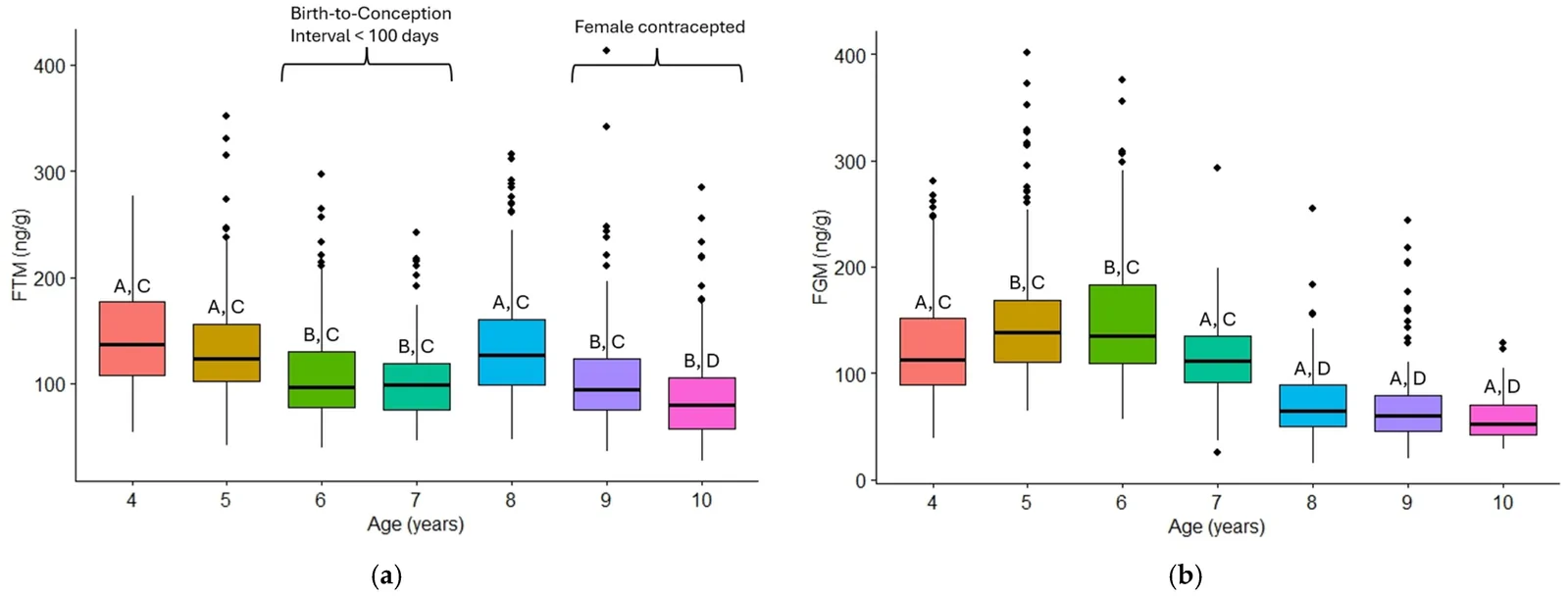
Changes in (**a**) FTM and (**b**) FGM concentrations for 1 adult male bontebok (Karoo) across several ages (in years). Ages with different letters (A, B, C or D) are significantly different from one another (*p* < 0.05). Box plots show the median (center line), interquartile range (box), 1.5× interquartile range (whiskers), and individual outliers (rhombus). Reproductive events for co-housed female is specified above ages for reference.

**Figure 11:**
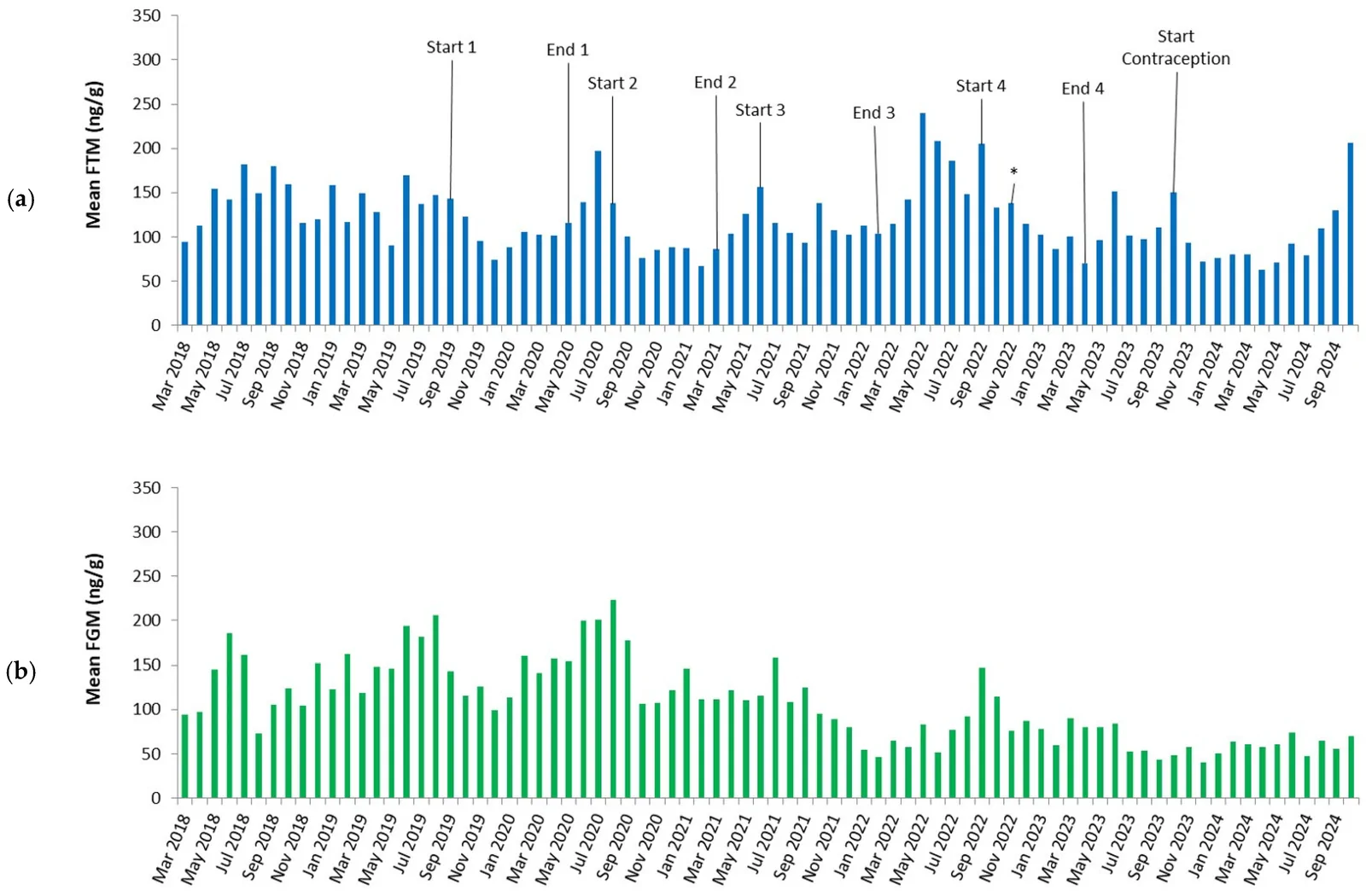
Longitudinal patterns in monthly (**a**) FTM and (**b**) FGM means in an adult male bontebok (Karoo) over a 6-year time span. “Start” and “End” denote the start and end of a pregnancy in the co-housed female, followed by a number to indicate if it was the start or end of the 1st, 2nd, 3rd, or 4th pregnancy or contraception. * = month that juvenile female started cycling.

When analyzing seasonal changes in FTM, the full ANOVA model showed that month and age were both significant factors (month: F (11, 1208) = 13.5, *p* < 0.001, *η*^2^ = 0.072; age: F (10, 1208) = 45.7, *p* < 0.001, *η*^2^ = 0.255) and this continued to be true even when we focused only on one male with several complete years of fecal sample collection (month: F (11, 1083) = 14.1, *p* < 0.001, *η*^2^ = 0.119; age: F (7, 1083) = 33.1, *p* < 0.001, *η*^2^ = 0.155). For this adult male (Karoo), we saw that FTM concentrations were significantly higher from June through October than during all other months (Figure 9a). Although we do not know exactly when the breeding season starts, these are four of the months in which a successful copulation was observed. We also saw an interesting difference in FTM concentrations between different ages in Karoo. According to Tukey’s HSD, Karoo’s FTM concentrations were highest at the ages of 4, 5, and 8 and lowest at ages 6, 7, 9 and 10 (Figure 10). In his longitudinal data (Figure 11), we observed a pattern that suggests Karoo’s FTM concentrations rise sharply after the female gives birth and decline soon after he has mated with the female.

For the FGM seasonal analysis, both month and age were significant factors in the 3-male ANOVA model (month: F (11, 1208) = 9.17, *p* < 0.001, *η*^2^ = 0.048; age: F (10, 1208) = 85.0, *p* < 0.001, *η*^2^ = 0.393) and the 1-male ANOVA model (month: F (11, 1083) = 6.44, *p* < 0.001, *η*^2^ = 0.034; age: F (7, 1083) = 143, *p* < 0.001, *η*^2^ = 0.463). Based on Tukey’s HSD, FGM concentrations were the highest in May, June and July (Figure 9) and Karoo had significantly higher FGM concentrations from ages 4 to 7 than ages 8 to 10 (Figure 10).

In addition, we found a significant, but weak, positive correlation between FTMs and FGMs (r (1229) = 0.346, *p* < 0.001). However, when we examined seasonal FTM and FGM patterns over time, we found that FTM concentrations remained elevated throughout the reproductive season, even when FGM concentrations started to drop (Figure 9). This suggests that FTMs and FGMs have different seasonal patterns even if they are weakly correlated.

## 4. Discussion

This study provides the first longitudinal data set on bontebok physiology, allowing detailed analyses of female and male hormonal changes over multiple years and reproductive states. Below we will highlight the main findings and their relative significance within the framework of the current literature.

### 4.1. Assay Validation

This study provides a preliminary validation of EIA assays for fecal progestagen, testosterone and glucocorticoid metabolites in both male and female bontebok. Fecal progestagen metabolite concentrations were shown to provide reliable monitoring of estrous cycles and pregnancy. Fecal testosterone metabolite concentrations were significantly different between juvenile and adult bontebok and repeatedly demonstrated seasonal changes. Fecal glucocorticoid metabolite concentrations increased significantly after known stressful events such as translocation or veterinary exams in both a male and female bontebok. However, these results are based on a limited number of animals, and thus may not provide a full representation of the variation in hormone values across the species, individuals, or physiological states.

Non-invasive fecal hormone monitoring has been applied to a range of related African antelope species in recent years, including two species in the same subfamily as bontebok (*Alcelphinae*): Jackson’s hartebeest (*Alcelaphus buselaphus jacksoni*) [26,27] and blue wildebeest (*Connochaetes taurinus*) [20,21,28,29], as well as numerous species in the same family (*Bovidae*), such as sable antelope (*Hippotragus niger*) [23], roan antelope (*Hippotragus equinus*) [30], gerenuk (*Litocranius walleri*) [31], scimitar-horned oryx (*Oryx dammah*) [32], mountain nyala (*Tragelaphus buxtoni*), impala (*Aepyceros melampus*), kudu (*Tragelaphus strepsiceros*) [29] and several species of gazelle [33,34,35,36,37,38]. To the best of our knowledge, this is the first assay validation publication for bontebok and therefore an important addition to the “library” of available antelope hormone assays for future monitoring needs.

### 4.2. Estrous Cycle Length and Gestation

The present study successfully used fecal progestagen metabolites to characterize estrous cycles and multiple pregnancies in female bontebok. Our data show that estrous cycles in female bontebok averaged 22.1 ± 0.29 days and ranged from 21 to 25 days. As far as we know, to date, no studies have described the estrous cycle length in bontebok or their closest relative, the blesbok (*Damaliscus pygargus phillipsi*). However, estrous cycle lengths have been characterized using endocrine methods for several species in the bontebok’s subfamily (*Alcelaphinae*) and the next closest subfamily (*Hippotraginae*). The bontebok’s estrous cycle length is almost the same as other species in their subfamily (tsessebe (*Damaliscus lunatus lunatus*), 18–23 days [39], blue wildebeest, 22.6 + 1.0 days [28], Jackson’s hartebeest, 21.4 ± 4.1 days (range 17–32) [26]), and is only a few days shorter than the estrous cycle reported for species in *Hippotraginae* (sable antelope, 24.2 ± 0.9 days [23], roan antelope, 30.8 ± 2.1 days [40], Arabian oryx, 23.7 ± 1.3 days (range: 21–26 days) [41], scimitar-horned oryx, 23.8 ± 1.3 days [32], and addax (*Addax nasomaculatus*), 22.7 ± 2.0 days [42]).

In this study, gestation length, as determined by sustained elevations in fecal progestagen metabolites in one female, ranged from 223 to 236 days. Based on behavioral observations of free-ranging bontebok, most published studies on bontebok report a gestation period of 7.5 to 8 months (approximately 225–240 days) [2,43,44], with one source estimating it to last as long as 254 days [45]. This range is quite comparable with our findings. In addition, the gestation length we observed for our bontebok aligns very closely with the range observed across other species in the subfamily *Alcelaphinae*. For the closest living relatives of the bontebok, the blesbok and the tsessebe, gestation has been behaviorally estimated to have the same range as bontebok (225–240 days) [46,47,48]. In the closely related hartebeest, blue wildebeest, and black wildebeest (*Connochaetes gnou*), gestation is generally reported to be 8–8.5 months (240–255 days), with some sources reporting it as long as 9 months (270 days) [28,43,49,50,51]. Across the *Alcelaphinae* subfamily, reported gestation lengths generally range from 7.5 to 9 months (225–270 days), and the bontebok gestation length documented in our study fits well within this range, although on the shorter end. By comparison, gestation lengths in the *Hippotraginae* tend to be slightly longer (sable antelope, 240–272 days [52,53]; roan antelope, 270–290 days [54]; addax (*Addax nasomaculatus*), 257–264 days [55]; gemsbok (*Oryx gazella*), 261–275 days [56]; and Arabian oryx (*Oryx leucoryx*), 255–273 days [41]).

However, all of these reported gestation lengths have been estimated using behavioral methods and not endocrine methods. Across these two subfamilies, endocrine methods have been used to estimate gestation length in only three species. In the blue wildebeest, Clay et al. (2010) used fecal progestagens to determine a mean gestation length of 240.8 ± 11.7 days (*n* = 4) [28]. In roan antelope, gestation length was estimated by Kamgang et al. (2023) using fecal progestagens as 280.4 ± 4.8 days (*n* = 10) [40]. And in the Arabian oryx, Sempéré et al. (1996) used serum progesterone to estimate gestation length as 255.2 ± 1.5 days (*n* = 13) [41]. Two of these endocrine-based estimates are on the lower end of each species’ reported ranges. These differences likely reflect methodological differences in determining conception dates (behavioral observation versus endocrine profiling), but inter-individual differences or the effects of captive management factors such as nutrition and reduced physical activity may also play a role in influencing gestation length estimates [57]. The estrous cycle and gestation lengths reported here provide the first endocrine-based estimates for this species, adding important baseline data for captive management and conservation breeding programs. In addition, these findings are consistent with the theory that this subspecies is seasonally polyestrous with spontaneous ovulation.

### 4.3. Reproductive Hormones and Breeding Season

Our findings indicate that male bontebok housed in the Northern Hemisphere showed peak fecal testosterone metabolite concentrations from June to October, with conceptions in our females occurring from June to September. These findings are partially consistent with observations of wild bontebok and related species in the Southern Hemisphere, but the dates appear to have shifted by 5–6 months.

For wild bontebok in the Southern Hemisphere, the breeding season occurs from January to March [2]. Skinner et al. (1980) reported that plasma testosterone in male bonteboks peaked in February, with the lowest concentrations occurring around June (winter in the Southern Hemisphere) [58]. However, Skinner et al. (1980) only analyzed plasma testosterone in 21 samples from 3 months (February, June and September) and, thus, a precise timing for the rise and fall in testosterone for this population is unknown. Nevertheless, the pattern is consistent with testosterone being elevated during the breeding season [58].

This approximately 6-month shift in reproductive timing between Southern Hemisphere wild populations and Northern Hemisphere zoo-housed populations is consistent with the potential role of photoperiod as a driver of reproductive seasonality in this species, as has been proposed for other seasonal ruminants [59]. Among the subfamily *Alcelaphinae*, significant correlations between day length and the timing of breeding seasons have been documented. Notably, both the tsessebe and the black wildebeest showed a similar approximately 6-month shift in birth seasons when comparing wild populations in the Southern Hemisphere with captive populations in the Northern Hemisphere [59]. These findings collectively indicate that photoperiodic cues are common within the *Alcelaphinae* and that bontebok may retain photoperiod-dependent reproductive regulation even when maintained under human care conditions.

However, the relationship between photoperiod and reproductive seasonality is not uniform across all bovid species. Among the *Hippotraginae*, some species appear to shift from strongly seasonal reproduction when free-ranging in their original habitat to non-seasonal or weakly seasonal reproduction in human care conditions. The sable antelope, for example, exhibits year-round estrous cyclicity when kept in North America, with no clear evidence of seasonal anestrus, despite having a defined breeding season (May–July) in wild southern African populations [23]. Similarly, the blackbuck (*Antilope cervicapra*) and several gazelle species (e.g., *Gazella* spp.) have been reported to transition from seasonal to non-seasonal breeding when maintained in human care under conditions of continuous food availability [59]. This transition suggests that, in these species, reproductive seasonality is controlled primarily by nutritional or body-condition thresholds rather than photoperiodic cues, and that the removal of resource limitation in confined settings unmasks an underlying capacity for year-round reproduction [59,60]. The retention of reproductive seasonality observed in bontebok under human care in various settings, despite ad libitum food availability, therefore distinguishes this species from resource-driven seasonal breeders and supports the hypothesis that bontebok possess a photoperiod-dependent timing mechanism for reproduction.

### 4.4. Seasonal Changes in Glucocorticoids

Both female and male bontebok exhibited significant seasonal variation in fecal glucocorticoid metabolite concentrations, with the highest values recorded in May, June and July. These months coincide with the onset of both the dry season in Oregon and the breeding season for bontebok maintained at Northern Hemisphere latitudes. Higher FGM concentrations in the dry season have been reported in many bovid species including the blue wildebeest [29,61], impala, nyala, kudu [29], roan antelope [30], banteng [62], blackbuck [63], and hartebeest [27], but the explanations for these seasonal trends vary.

There are several possible explanations for seasonal trends in FGMs: (1) seasonal changes in resource availability and nutritional status, (2) predictable changes in energetic demands for thermoregulation or reproductive-related events, and (3) cyclical changes in predation pressure and social stress [64,65]. A particular strength of the present study is that it was conducted on a zoo-housed population in which the determinants most often invoked to explain seasonal FGM variation in free-ranging ungulates—forage quantity and quality, drinking water availability, predation pressure, and the composition and density of the social group—were relatively constant throughout the year. This suggests that seasonal variation in FGM concentrations in bontebok is probably associated with energy mobilization for either thermoregulation or preparation for the breeding season.

Glucocorticoids rise predictably during energetically demanding reproductive events such as courtship, territorial defense, gestation and lactation, and several bovids show comparable patterns [64]. For example, captive female Jackson’s hartebeest had higher FGM concentrations from April to July and during pregnancy, a pattern attributed to seasonal environmental factors and mating-related events [27]. In captive roan antelope, males exhibited elevated FGMs during periods of active reproduction [30], and in zoo-housed banteng (*Bos javanicus*), both FGMs and androgen hormone output were higher during the summer breeding months [62].

Direct environmental exposure and the metabolic cost of thermoregulation offer a second, non-exclusive explanation for seasonal FGM patterns, as the May–July peak corresponds to the warmest, longest-photoperiod part of the year. For example, captive blackbuck had higher glucocorticoids in summer than in winter, with glucocorticoids positively correlating with temperature and negatively correlating with humidity [63]. Captive roan antelope and banteng also showed higher FGMs in the dry, warmer season [30,62]. Conversely, some ungulates show the opposite pattern with reduced adrenocortical activity during the breeding season or dry season [66,67]. Pyrenean chamois (*Rupicapra pyrenaica*) and scimitar-horned oryx displayed peak FGMs during the winter in association with the energetic demands of cold [67] and chital deer (*Axis axis*) had lower serum glucocorticoids during the breeding season [66]. Przewalski’s gazelle (*Procapra przewalskii*) displayed its highest FGM concentrations during the lactation period and its lowest concentrations during the breeding season, implying that the lactational rather than the mating phase imposed the greater allostatic load [35]. Because temperature, rainfall and photoperiod co-vary seasonally and could not be experimentally separated here, the present data cannot distinguish a direct thermal-load mechanism from a photoperiod-cued endocrine response.

In contrast, two explanations that frequently account for seasonal FGM variation in the wild are unlikely to apply to the present zoo-housed population. The first is seasonal change in resource availability and nutritional status. The relationship between FGM concentrations and nutritional status was shown clearly in American plains bison (*Bison bison*), in which seasonal changes in FGMs tracked dietary protein and fiber content and the seasonal pattern was eliminated once animals were transitioned to a consistent grain and hay diet [68]. In free-ranging populations, deteriorating forage is among the most common correlates of elevated FGM. For example, white-bearded wildebeest (*Connochaetes taurinus*) had higher dry season FGMs that declined as vegetation greenness increased [61], and increased dry season elevations have been described in several wild ungulates including impala, greater kudu, and mountain nyala [29].

The second unlikely explanation is cyclical variation in predation risk or social stress. High predation risk produces stress in some prey species, and this may vary seasonally if predator density changes seasonally or prey are more vulnerable during different seasons [65,69,70]. For example, predators may be more likely to target mothers with newborns during the calving season [37]. Increased predation pressure has been linked to increased FGMs in some species like the snowshoe hare (*Lepus americanus*), but based on his review, Boonstra (2013) suggested that the predation pressure on longer-lived ungulates, such as elk, is better explained by changes in foraging patterns that carry nutritional costs than by changes in FGM concentrations [65,70]. This has been shown to be true in other ungulate species. For example, impala and blue wildebeest exposed to differing intensities of lion (*Panthera leo*) presence showed no corresponding variation in FGMs [22]. And bison populations exposed to wolf (*Canis lupus*) predation showed a different seasonal pattern in FGMs than populations without wolves, but the inconsistency of the pattern led authors to suggest that FGM changes in bison were attributed to seasonal changes in energy demand, and not predation pressure [71].

A possible consideration in zoo-housed species is that visitor numbers vary seasonally, with increased attendance and visitor-focused activities in the summer. Some zoo-housed prey species may react to the increased presence of humans as a form of predator pressure [72]. For example, Karaer et al. (2024) found that captive mountain gazelles (*Gazella gazella*) exhibited significantly higher FGMs in September, when the visitor numbers were at their peak, than in other months of the year, and they also showed higher FGMs than free-ranging mountain gazelles [37]. While we did observe a rise in FGM concentrations in the summer for bontebok in general, there is no evidence that it was related to visitor presence. For example, in 2020, when no visitors were allowed due to COVID-19 restrictions, bontebok FGM concentrations in May, June and July of 2020 were actually the same or higher than in other years. This suggests visitor presence was not directly linked to higher FGMs in bontebok.

Social context, such as the density and composition of social groups, can also influence FGM concentrations. Zoo-housed Cuvier’s gazelle (*Gazella cuvieri*) males had higher FGM concentrations when housed alone than with other males, whereas Dorcas gazelle (*Gazella dorcas*) and Mohor gazelle (*Nanger dama mhorr*) males had lower FGM concentrations when housed alone [73]. Because natural predators were absent and the social environment was held relatively constant in the present study, neither predation risk nor seasonally shifting social pressure offers a good explanation for the observed seasonal pattern.

In summary, a clear seasonal FGM pattern in a setting where nutrition and water availability, predation pressure and social composition remained constant points to photoperiod, thermal load and reproductive physiology as the most plausible drivers of seasonal hormonal patterns. Ultimately, the FGM data provided in this study can serve as a useful baseline for future animal well-being monitoring in bontebok and for interpreting glucocorticoid data in other, closely related ungulates under human care.

### 4.5. Caveats and Future Work

A limitation of this study is the very small sample size of individuals for which complete estrous cycles, pregnancies and seasonal data were obtained. Because all pregnancies were monitored in the same female, we cannot say whether this hormone pattern and gestation length hold true for the whole species, but this can provide a starting point for estimating gestation patterns in other female bontebok. Additionally, the precise onset and cessation of the breeding season may vary among individuals and across years due to factors such as age, body condition, and social dynamics. While we saw consistent seasonal hormone patterns within both our male and female bontebok from year to year, we cannot say whether this applies to the whole species. Future work would benefit from multi-year, multi-institutional sampling across a range of latitudes, to determine whether the degree of seasonal shift is consistent and whether daylength at conception is conserved, as predicted by the photoperiodic control hypothesis.

## 5. Conclusions

This study provides the first validation of non-invasive endocrine methods and longitudinal hormone profiles for the bontebok. Enzyme immunoassays for fecal progestagen, testosterone, and glucocorticoid metabolites were successfully validated in both sexes, with glucocorticoid concentrations rising sharply after stressful events such as inter-facility transfers and veterinary examinations. Progestagen monitoring revealed an estrous cycle of approximately 21 to 25 days and a gestation length of roughly 223 to 236 days across four pregnancies, with progestagen concentrations declining shortly before parturition. Male testosterone metabolites peaked from June to October, coinciding with observed conceptions, while females cycled from September to January. This shows that testosterone is elevated during the breeding season and that there is an approximately six-month shift in the breeding season relative to wild Southern Hemisphere populations, likely due to photoperiodic inversion. Both sexes showed seasonal peaks in glucocorticoid metabolites during late spring and early summer, even though food, water, and social conditions remained constant, suggesting these patterns reflect thermoregulatory and reproductive demands rather than resource limitation or predation pressure. Taken together, these results support the theory that bontebok are a seasonal, polyestrous, spontaneously ovulating subspecies and provide a valuable endocrine baseline to guide reproductive management, welfare assessments, and conservation breeding for the population recovery of this African antelope.

## Figures and Tables

**Table 1:** Background and fecal collection information about each study animal.

Animal Name	Studbook	Birthdate	Sex	Sample Size	Fecal Collection
May	460	2 May 2012	Female	26	October 2025–January 2026 (3 months)
Karoo	471	15 June 2014	Male	1201	March 2018–May 2025 (7+ years)
Bhisho	472	21 June 2014	Male	52	February 2016–May 2016 (4 months)
Winter	479	28 May 2015	Female	1199	June 2018–January 2026 (7+ years)
Tutula	556	2 February 2022	Female	255	May 2022–May 2024 (2 years)
Monte	590	1 April 2023	Male	76	May 2023–December 2023 (8 months)

**Table 2:** Comparison of FPM and FGM means (and medians) for all females during different reproductive stages.

Reproductive Phase	FPM Mean ± SE (Median)	FGM Mean ± SE (Median)	*n* *
Contracepted	112 ± 1.85 (107)	69.3 ± 2.42 (57.2)	342
Juvenile	123 ± 12.3 (105)	82.8 ± 6.24 (77.9)	42
Adult, non-breeding	146 ± 2.38 (135)	124 ± 3.23 (108)	532
Cycling	539 ± 20.9 (553)	120 ± 4.30 (110)	162
Pregnancy	738 ± 8.90 (734)	174 ± 4.02 (151)	402

* *n* = sample size, FPMs = fecal progestagen metabolites, FGMs = fecal glucocorticoid metabolites, SE = standard error; all steroid values are in ng/g.

**Table 3:** Overall, baseline and luteal means for FPMs and overall and baseline means for FGMs for each female bontebok. Medians were provided for overall FPM, luteal FPM and overall FGM concentrations. Standard errors (SEs) were provided for overall and luteal means and elevated thresholds were provided for baseline means.

Animal	Winter	Tutula	May
Overall FPM Mean ± SE (median)	386 ± 9.01 (186)	148 ± 10.9 (102)	446 ± 45.5 (516)
Baseline FPM Mean/Threshold	172/345	106/345 *	160/345 *
Luteal FPM ± SE (median, *n*)	651 ± 19.4 (633, *n* = 99)	783 ± 60.9 (766, *n* = 15)	561 ± 34.1 (629, *n* = 19)
Overall FGM Mean ± SE (median)	135 ± 2.29 (127)	69.8 ± 2.34 (60.2)	97.7 ± 4.45 (91.4)
Baseline FGM Mean/Threshold	87.8/170	53.5/103	89.4/110

* Due to limited sample size, Winter’s threshold was used to identify elevated luteal phase samples in these females. *n* = number of luteal phase samples analyzed. All steroid values are in ng/g.

**Table 4:** Estimated dates of conception and parturition, gestation length (# days), FPM means and medians and FGM means and medians during early, middle and late gestation in an adult female bontebok (Winter, #479) for 4 pregnancies. All steroid values are in ng/g. Sample size is the same for both FGM and FPM means.

				Sample Size			FPM Mean (Median)			FGM Mean (Median)		
Pregnancy	Estimated Conception	Parturition Date	# Days	Early	Mid	Late	Early	Middle	Late	Early	Middle	Late
1	30 September 2019	16 May 2020	229	43	42	42	784 (787)	823 (773)	650 (642)	171 (156)	177 (164)	295 (262)
2	25 July 2020	16 March 2021	234	27	37	45	891 (916)	862 (857)	604 (587)	235 (236)	178 (179)	177 (156)
3	24 June 2021	2 February 2022	223	12	37	49	791 (808)	754 (738)	692 (654)	152 (152)	136 (129)	121 (109)
4	10 August 2022	3 April 2023	236	7	27	33	787 (789)	758 (769)	616 (606)	154 (133)	112 (114)	133 (127)
	Mean (Median)		231				818 (839)	803 (773)	643 (634)	187 (171)	154 (144)	182 (148)

**Table 5:** Overall means ± SE (medians) and baseline means/elevated thresholds for FTMs and FGMs for each male bontebok.

Animal	Karoo	Bhisho	Monte
Overall FTM * Mean ± SE (median)	138 ± 4.58 (109)	77.5 ± 5.27 (61.1)	163 ± 7.76 (148)
Baseline FTM Mean/Threshold	103/163	65.6/132	120/140
Overall FGM Mean ± SE (median)	138 ± 4.07 (102)	90.9 ± 6.73 (71.8)	330 ± 50.3 (108)
Baseline FGM Mean/Threshold	92.1/165	64.0/114	80.8/118

* FTMs = fecal testosterone metabolites, FGMs = fecal glucocorticoid metabolites. All steroid values are in ng/g.

## Data Availability

The data presented in this study are available on request from the corresponding author. The data are not publicly available due to the confidential nature of health data at the Oregon Zoo.

## Supplementary Materials

The following supporting information can be downloaded at: https://www.mdpi.com/article/10.3390/ani16203171/s1, Table S1: ANOVA results for each of 12 models mentioned in the results including specifics about how many animals and samples were included in the data set. The “variable” chart for each model includes the sum of squares (Sum Sq), degrees of freedom (df), F-values (F), *p*-values and effect size (*η*^2^) for each variable. Subsequent charts include between group differences, upper and lower limits of the 95% confidence level and *p*-values for categories within each variable according to Tukey’s HSD.

## Abbreviations

The following abbreviations are used in this manuscript:

EIA	Enzyme immunoassay
FGM	Fecal glucocorticoid metabolite
FPM	Fecal progestagen metabolite
FTM	Fecal testosterone metabolite
SD	Standard deviation
SE	Standard error
