# Supplementary material for: Longitudinal Analysis of Reproductive and Adrenal Hormones in Bontebok (Damaliscus pygargus pygargus)
Source: Animals (Basel). 2026 Oct 9;16(20):3171. doi: 10.3390/ani16203171 (PMC13700830; doi:10.3390/ani16203171)
Supplement: Supplementary file 1 [file animals-16-03171-s001.zip › animals-4498107-supplementary.pdf]

Table S1. ANOVA results for each of 12 models mentioned in the results including specifics about how many animals and samples were included in the data set. The "variable" chart for each model includes the sum of squares (Sum Sq), degrees of freedom (df), F-values (F), *p*-values and effect size (η^2) for each variable. Subsequent charts include between group differences, upper and lower limits of the 95% confidence level and *p*-values for categories within each variable according to Tukey's HSD.

Model 1: aov (log FPM ~ Reproductive Stage, data = 3 females, number of samples = 1477)

Variable	Sum Sq	df	F	*p*-value	η^2
Reproductive Stage	178.414	4	1879	<0.001	0.836
Residuals	34.943	1472			

Reproductive Stage	Difference	Lower Limit	Upper Limit	*p*-value
Cycling Adult - Contracepted	0.623	0.584	0.662	<0.001
Juvenile - Contracepted	0.016	-0.052	0.085	0.966
Non-Pregnant Adult - Contracepted	0.109	0.080	0.138	<0.001
Pregnant - Contracepted	0.821	0.790	0.852	<0.001
Juvenile - Cycling Adult	-0.607	-0.679	-0.535	<0.001
Non-Pregnant Adult - Cycling	-0.514	-0.551	-0.478	<0.001
Pregnant - Cycling Adult	0.197	0.160	0.235	<0.001
Non-Pregnant Adult - Juvenile	0.093	0.025	0.160	0.002
Pregnant - Juvenile	0.804	0.736	0.872	<0.001
Pregnant - Non-Pregnant Adult	0.711	0.683	0.740	<0.001

Model 2: aov (log FPM ~ Gestational Period + Pregnancy Number, data = 4 pregnancies in 1 female, number of samples = 402)

Variable	Sum Sq	df	F	*p*-value	η^2
Gestational Period	0.970	2	40.2	<0.001	0.172
Pregnancy Number	0.037	3	1.03	0.379	0.006
Residuals	4.78	396			

Gestational Period	Difference	Lower Limit	Upper Limit	*p*-value
Late-Early	-0.105	-0.139	-0.072	0.000
Middle-Early	-0.008	-0.043	0.027	0.860
Middle-Late	0.098	0.068	0.127	0.000

Pregnancy Number	Difference	Lower Limit	Upper Limit	*p*-value
Preg2-Preg1	0.000	-0.036	0.037	1.000

Preg3-Preg1	0.010	-0.028	0.048	0.915
Preg4-Preg1	-0.021	-0.063	0.022	0.598
Preg3-Preg2	0.009	-0.030	0.049	0.933
Preg4-Preg2	-0.021	-0.065	0.023	0.603
Preg4-Preg3	-0.030	-0.075	0.015	0.306

Model 3: aov (log FGM ~ Gestational Period + Pregnancy Number, data = 4 pregnancies in 1 female, number of samples = 402)

Variable	Sum Sq	df	F	*p*-value	η^2
Gestational Period	0.225	2	4.75	0.009	0.032
Pregnancy Number	3.50	3	49.3	<0.001	0.263
Residuals	9.36	396			

Gestational Period	Difference	Lower Limit	Upper Limit	*p*-value
Late-Early	-0.041	-0.088	0.006	0.104
Middle-Early	-0.086	-0.135	-0.038	<0.001
Middle-Late	-0.045	-0.086	-0.004	0.026

Pregnancy Number	Difference	Lower Limit	Upper Limit	*p*-value
Preg2-Preg1	-0.030	-0.081	0.022	0.453
Preg3-Preg1	-0.198	-0.251	-0.144	<0.001
Preg4-Preg1	-0.196	-0.256	-0.136	<0.001
Preg3-Preg2	-0.168	-0.223	-0.113	<0.001
Preg4-Preg2	-0.166	-0.228	-0.105	<0.001
Preg4-Preg3	0.002	-0.061	0.064	1.000

Model 4: aov (log FPM ~ Month + Age + Reproductive Stage, data = 2 females, number of samples = 1451)

Variable	Sum Sq	df	F	*p*-value	η^2
Month	0.691	11	3.11	<0.001	0.241
Age (year)	2.92	11	13.1	<0.001	0.368
Reproductive Stage	53.4	4	661	<0.001	0.254
Residuals	28.7	1424			

Reproductive Stage	Difference	Lower Limit	Upper Limit	*p*-value
Cycling Adult - Contracepted	0.138	0.100	0.176	<0.001

Juvenile - Contracepted	-0.090	-0.154	-0.027	<0.001
Non-Pregnant Adult - Contracepted	-0.104	-0.131	-0.077	<0.001
Pregnant - Contracepted	0.180	0.152	0.209	<0.001
Juvenile - Cycling Adult	-0.228	-0.296	-0.161	<0.001
Non-Pregnant Adult - Cycling	-0.242	-0.277	-0.206	<0.001
Pregnant - Cycling Adult	0.042	0.005	0.079	0.015
Non-Pregnant Adult - Juvenile	-0.013	-0.076	0.049	0.977
Pregnant - Juvenile	0.270	0.208	0.333	<0.001
Pregnant - Non-Pregnant Adult	0.284	0.258	0.310	<0.001

Model 5: aov (log FPM ~ Month + Age + Reproductive Stage, data = 2 females, no pregnancy data, number of samples = 1049)

Variable	Sum Sq	df	F	*p*-value	η^2
Month	0.534	11	2.17	0.014	0.178
Age (year)	3.14	11	12.7	<0.001	0.264
Reproductive Stage	17.5	3	261	<0.001	0.242
Residuals	22.9	1023			

Month	Difference	Lower Limit	Upper Limit	*p*-value
2-1	-0.230	-0.315	-0.145	<0.001
3-1	-0.268	-0.348	-0.188	<0.001
4-1	-0.237	-0.315	-0.159	<0.001
5-1	-0.217	-0.295	-0.139	<0.001
6-1	-0.204	-0.281	-0.128	<0.001
7-1	-0.228	-0.306	-0.150	<0.001
8-1	-0.226	-0.306	-0.145	<0.001
9-1	-0.042	-0.125	0.040	0.877
10-1	0.046	-0.041	0.133	0.858
11-1	-0.029	-0.115	0.057	0.995
12-1	0.035	-0.051	0.120	0.975
3-2	-0.038	-0.114	0.039	0.908
4-2	-0.007	-0.081	0.068	1.000
5-2	0.013	-0.061	0.088	1.000
6-2	0.026	-0.047	0.099	0.991
7-2	0.002	-0.073	0.077	1.000
8-2	0.005	-0.073	0.082	1.000
9-2	0.188	0.109	0.267	<0.001
10-2	0.276	0.192	0.360	<0.001
11-2	0.201	0.118	0.284	<0.001

12-2	0.265	0.183	0.347	<0.001
4-3	0.031	-0.038	0.100	0.952
5-3	0.051	-0.018	0.120	0.399
6-3	0.064	-0.004	0.131	0.088
7-3	0.040	-0.030	0.109	0.771
8-3	0.042	-0.030	0.114	0.748
9-3	0.226	0.151	0.300	<0.001
10-3	0.314	0.234	0.393	<0.001
11-3	0.239	0.161	0.317	<0.001
12-3	0.303	0.225	0.380	<0.001
5-4	0.020	-0.047	0.087	0.998
6-4	0.033	-0.032	0.098	0.889
7-4	0.009	-0.058	0.076	1.000
8-4	0.011	-0.058	0.081	1.000
9-4	0.195	0.123	0.267	<0.001
10-4	0.283	0.206	0.360	<0.001
11-4	0.208	0.132	0.284	<0.001
12-4	0.272	0.197	0.347	<0.001
6-5	0.013	-0.052	0.078	1.000
7-5	-0.011	-0.078	0.056	1.000
8-5	-0.009	-0.078	0.061	1.000
9-5	0.175	0.103	0.246	<0.001
10-5	0.263	0.186	0.340	<0.001
11-5	0.188	0.112	0.264	<0.001
12-5	0.252	0.177	0.327	<0.001
7-6	-0.024	-0.089	0.042	0.990
8-6	-0.021	-0.089	0.046	0.997
9-6	0.162	0.092	0.232	<0.001
10-6	0.250	0.175	0.326	<0.001
11-6	0.175	0.101	0.250	<0.001
12-6	0.239	0.166	0.312	<0.001
8-7	0.002	-0.068	0.072	1.000
9-7	0.186	0.114	0.258	<0.001
10-7	0.274	0.196	0.351	<0.001
11-7	0.199	0.123	0.275	<0.001
12-7	0.263	0.188	0.338	<0.001
9-8	0.183	0.109	0.258	<0.001
10-8	0.271	0.192	0.351	<0.001
11-8	0.197	0.118	0.275	<0.001
12-8	0.260	0.183	0.338	<0.001
10-9	0.088	0.007	0.169	0.052
11-9	0.013	-0.067	0.094	1.000

12-9	0.077	-0.002	0.156	0.068
11-10	-0.075	-0.160	0.010	0.151
12-10	-0.011	-0.095	0.073	1.000
12-11	0.064	-0.020	0.147	0.337

Age (year)	Difference	Lower Limit	Upper Limit	*p*-value
1-0	-0.033	-0.237	0.171	1.000
2-0	-0.210	-0.415	-0.004	0.040
3-0	0.145	-0.061	0.350	0.475
4-0	0.210	0.007	0.414	0.035
5-0	0.056	-0.166	0.278	1.000
6-0	0.097	-0.114	0.308	0.938
7-0	0.001	-0.206	0.208	1.000
8-0	-0.018	-0.224	0.189	1.000
9-0	-0.107	-0.313	0.098	0.863
10-0	-0.021	-0.227	0.185	1.000
11-0	0.294	0.066	0.522	0.002
2-1	-0.177	-0.239	-0.114	<0.001
3-1	0.178	0.115	0.241	<0.001
4-1	0.243	0.188	0.298	<0.001
5-1	0.089	-0.016	0.194	0.186
6-1	0.130	0.052	0.209	<0.001
7-1	0.034	-0.033	0.101	0.893
8-1	0.015	-0.050	0.081	1.000
9-1	-0.074	-0.137	-0.011	0.006
10-1	0.012	-0.051	0.075	1.000
11-1	0.327	0.210	0.444	<0.001
3-2	0.354	0.288	0.421	<0.001
4-2	0.420	0.361	0.479	<0.001
5-2	0.266	0.159	0.373	<0.001
6-2	0.307	0.226	0.389	<0.001
7-2	0.211	0.140	0.281	<0.001
8-2	0.192	0.124	0.261	<0.001
9-2	0.103	0.036	0.169	<0.001
10-2	0.189	0.123	0.255	<0.001
11-2	0.504	0.385	0.623	<0.001
4-3	0.066	0.006	0.125	0.015
5-3	-0.088	-0.195	0.019	0.228
6-3	-0.047	-0.129	0.034	0.762
7-3	-0.144	-0.215	-0.073	<0.001
8-3	-0.162	-0.231	-0.093	<0.001

9-3	-0.252	-0.319	-0.185	<0.001
10-3	-0.166	-0.232	-0.099	<0.001
11-3	0.149	0.030	0.269	0.003
5-4	-0.154	-0.256	-0.051	<0.001
6-4	-0.113	-0.189	-0.037	<0.001
7-4	-0.210	-0.273	-0.146	<0.001
8-4	-0.228	-0.290	-0.166	<0.001
9-4	-0.318	-0.377	-0.258	<0.001
10-4	-0.231	-0.290	-0.172	<0.001
11-4	0.084	-0.032	0.199	0.420
6-5	0.041	-0.076	0.158	0.992
7-5	-0.056	-0.165	0.054	0.884
8-5	-0.074	-0.183	0.034	0.524
9-5	-0.164	-0.271	-0.057	<0.001
10-5	-0.077	-0.184	0.030	0.432
11-5	0.238	0.092	0.383	<0.001
7-6	-0.097	-0.182	-0.012	0.011
8-6	-0.115	-0.199	-0.031	<0.001
9-6	-0.205	-0.286	-0.123	<0.001
10-6	-0.118	-0.200	-0.037	<0.001
11-6	0.197	0.068	0.325	<0.001
8-7	-0.018	-0.091	0.054	1.000
9-7	-0.108	-0.179	-0.037	<0.001
10-7	-0.022	-0.092	0.049	0.998
11-7	0.293	0.172	0.415	<0.001
9-8	-0.090	-0.159	-0.021	0.001
10-8	-0.003	-0.072	0.066	1.000
11-8	0.312	0.191	0.432	<0.001
10-9	0.086	0.020	0.153	0.001
11-9	0.401	0.282	0.521	<0.001
11-10	0.315	0.196	0.434	<0.001

Reproductive Stage	Difference	Lower Limit	Upper Limit	*p*-value
Cycling Adult - Contracepted	0.232	0.194	0.269	<0.001
Juvenile - Contracepted	-0.070	-0.133	-0.007	0.022
Non-Pregnant Adult - Contracepted	-0.008	-0.035	0.019	0.859
Juvenile - Cycling Adult	-0.302	-0.369	-0.235	<0.001
Non-Pregnant Adult - Cycling Adult	-0.240	-0.275	-0.204	<0.001
Non-Pregnant Adult - Juvenile	0.062	0.000	0.124	0.050

Model 6: aov (log FGM ~ Month + Age + Reproductive Stage, data = 2 females, number of samples = 1451)

Variable	Sum Sq	df	F	*p*-value	η^2
Month	2.79	11	8.68	<0.001	0.009
Age (year)	28.5	11	88.7	<0.001	0.545
Reproductive Stage	6.50	4	55.6	<0.001	0.060
Residuals	41.6	1424			

Reproductive Stage	Difference	Lower Limit	Upper Limit	*p*-value
Cycling Adult - Contracepted	-0.007	-0.052	0.039	0.995
Juvenile - Contracepted	0.034	-0.043	0.110	0.746
Non-Pregnant Adult - Contracepted	-0.039	-0.071	-0.006	0.011
Pregnant - Contracepted	0.070	0.036	0.105	<0.001
Juvenile - Cycling Adult	0.040	-0.041	0.122	0.657
Non-Pregnant Adult - Cycling	-0.032	-0.075	0.011	0.248
Pregnant - Cycling Adult	0.077	0.032	0.121	<0.001
Non-Pregnant Adult - Juvenile	-0.072	-0.147	0.003	0.064
Pregnant - Juvenile	0.036	-0.039	0.112	0.682
Pregnant - Non-Pregnant Adult	0.109	0.078	0.140	<0.001

Model 7: aov (logFGM ~ Month + Age + Reproductive Stage, data = 2 females, pregnancy data excluded, number of samples = 1049)

Variable	Sum Sq	df	F	*p*-value	η^2
Month	1.35	11	4.01	<0.001	0.046
Age (year)	27.9	11	82.5	<0.001	0.529
Reproductive Stage	0.253	3	2.75	0.052	0.003
Residuals	31.4	1023			

Month	Difference	Lower Limit	Upper Limit	*p*-value
2-1	-0.023	-0.122	0.077	1.000
3-1	-0.020	-0.114	0.074	1.000
4-1	0.008	-0.084	0.099	1.000
5-1	0.103	0.012	0.195	0.012
6-1	0.134	0.044	0.224	<0.001
7-1	0.032	-0.060	0.124	0.993
8-1	0.033	-0.061	0.128	0.992
9-1	0.037	-0.060	0.133	0.986

10-1	-0.010	-0.111	0.092	1.000
11-1	-0.032	-0.133	0.069	0.997
12-1	-0.066	-0.166	0.034	0.573
3-2	0.003	-0.087	0.093	1.000
4-2	0.031	-0.057	0.118	0.992
5-2	0.126	0.039	0.213	<0.001
6-2	0.157	0.071	0.242	<0.001
7-2	0.054	-0.033	0.142	0.671
8-2	0.056	-0.034	0.146	0.672
9-2	0.059	-0.034	0.152	0.631
10-2	0.013	-0.085	0.111	1.000
11-2	-0.009	-0.107	0.088	1.000
12-2	-0.043	-0.139	0.053	0.945
4-3	0.028	-0.053	0.109	0.994
5-3	0.123	0.042	0.204	<0.001
6-3	0.154	0.075	0.233	<0.001
7-3	0.051	-0.030	0.133	0.644
8-3	0.053	-0.031	0.137	0.651
9-3	0.056	-0.031	0.143	0.609
10-3	0.010	-0.083	0.103	1.000
11-3	-0.012	-0.104	0.079	1.000
12-3	-0.046	-0.137	0.044	0.874
5-4	0.095	0.017	0.173	0.004
6-4	0.126	0.050	0.202	<0.001
7-4	0.024	-0.055	0.102	0.998
8-4	0.025	-0.056	0.107	0.997
9-4	0.029	-0.056	0.113	0.994
10-4	-0.017	-0.108	0.073	1.000
11-4	-0.040	-0.129	0.049	0.947
12-4	-0.074	-0.162	0.014	0.197
6-5	0.031	-0.045	0.107	0.975
7-5	-0.071	-0.150	0.007	0.113
8-5	-0.070	-0.151	0.011	0.174
9-5	-0.067	-0.151	0.017	0.278
10-5	-0.113	-0.203	-0.023	0.003
11-5	-0.135	-0.224	-0.047	<0.001
12-5	-0.169	-0.257	-0.082	<0.001
7-6	-0.102	-0.179	-0.026	<0.001
8-6	-0.101	-0.180	-0.021	0.002
9-6	-0.098	-0.180	-0.015	0.006
10-6	-0.144	-0.232	-0.055	<0.001
11-6	-0.166	-0.253	-0.079	<0.001

12-6	-0.200	-0.286	-0.114	<0.001
8-7	0.002	-0.080	0.083	1.000
9-7	0.005	-0.080	0.089	1.000
10-7	-0.041	-0.132	0.049	0.942
11-7	-0.064	-0.153	0.025	0.445
12-7	-0.098	-0.186	-0.010	0.015
9-8	0.003	-0.084	0.090	1.000
10-8	-0.043	-0.136	0.050	0.938
11-8	-0.065	-0.157	0.026	0.453
12-8	-0.099	-0.190	-0.009	0.018
10-9	-0.046	-0.141	0.049	0.916
11-9	-0.069	-0.163	0.026	0.417
12-9	-0.103	-0.196	-0.010	0.017
11-10	-0.023	-0.122	0.077	1.000
12-10	-0.057	-0.155	0.042	0.772
12-11	-0.034	-0.131	0.063	0.993

Age (year)	Difference	Lower Limit	Upper Limit	*p*-value
1-0	0.020	-0.220	0.259	1.000
2-0	-0.124	-0.365	0.117	0.873
3-0	0.274	0.033	0.514	0.011
4-0	0.387	0.148	0.625	<0.001
5-0	0.331	0.071	0.591	0.002
6-0	0.235	-0.012	0.482	0.080
7-0	-0.099	-0.342	0.143	0.974
8-0	-0.092	-0.334	0.149	0.984
9-0	-0.024	-0.264	0.217	1.000
10-0	-0.046	-0.287	0.195	1.000
11-0	-0.025	-0.292	0.242	1.000
2-1	-0.144	-0.217	-0.071	<0.001
3-1	0.254	0.180	0.328	<0.001
4-1	0.367	0.303	0.431	<0.001
5-1	0.311	0.188	0.434	<0.001
6-1	0.215	0.123	0.308	<0.001
7-1	-0.119	-0.197	-0.040	<0.001
8-1	-0.112	-0.189	-0.036	<0.001
9-1	-0.043	-0.117	0.031	0.749
10-1	-0.065	-0.139	0.008	0.139
11-1	-0.045	-0.182	0.093	0.996
3-2	0.398	0.320	0.475	<0.001
4-2	0.511	0.442	0.579	<0.001

5-2	0.455	0.330	0.580	<0.001
6-2	0.359	0.264	0.454	<0.001
7-2	0.025	-0.057	0.107	0.998
8-2	0.032	-0.049	0.112	0.981
9-2	0.101	0.023	0.178	0.001
10-2	0.078	0.001	0.156	0.046
11-2	0.099	-0.040	0.239	0.456
4-3	0.113	0.044	0.182	<0.001
5-3	0.057	-0.068	0.183	0.942
6-3	-0.039	-0.134	0.057	0.976
7-3	-0.373	-0.456	-0.290	<0.001
8-3	-0.366	-0.447	-0.285	<0.001
9-3	-0.297	-0.375	-0.219	<0.001
10-3	-0.320	-0.398	-0.241	<0.001
11-3	-0.299	-0.438	-0.159	<0.001
5-4	-0.056	-0.176	0.064	0.936
6-4	-0.151	-0.240	-0.063	<0.001
7-4	-0.486	-0.560	-0.411	<0.001
8-4	-0.479	-0.551	-0.407	<0.001
9-4	-0.410	-0.479	-0.341	<0.001
10-4	-0.432	-0.502	-0.363	<0.001
11-4	-0.411	-0.546	-0.276	<0.001
6-5	-0.096	-0.233	0.041	0.484
7-5	-0.430	-0.558	-0.302	<0.001
8-5	-0.423	-0.550	-0.296	<0.001
9-5	-0.354	-0.480	-0.229	<0.001
10-5	-0.377	-0.502	-0.251	<0.001
11-5	-0.356	-0.526	-0.185	<0.001
7-6	-0.334	-0.434	-0.235	<0.001
8-6	-0.328	-0.425	-0.230	<0.001
9-6	-0.259	-0.354	-0.163	<0.001
10-6	-0.281	-0.377	-0.185	<0.001
11-6	-0.260	-0.410	-0.110	<0.001
8-7	0.007	-0.079	0.092	1.000
9-7	0.076	-0.007	0.158	0.111
10-7	0.053	-0.029	0.136	0.614
11-7	0.074	-0.068	0.217	0.864
9-8	0.069	-0.012	0.150	0.182
10-8	0.047	-0.034	0.127	0.764
11-8	0.068	-0.074	0.209	0.920
10-9	-0.022	-0.100	0.056	0.999
11-9	-0.001	-0.141	0.138	1.000

11-10	0.021	-0.119	0.161	1.000

Reproductive Stage	Difference	Lower Limit	Upper Limit	*p*-value
Cycling Adult - Contracepted	0.026	-0.018	0.070	0.431
Juvenile - Contracepted	0.026	-0.048	0.100	0.805
Non-Pregnant Adult - Contracepted	0.000	-0.032	0.031	1.000
Juvenile - Cycling Adult	0.000	-0.079	0.079	1.000
Non-Pregnant Adult - Cycling Adult	-0.026	-0.068	0.015	0.370
Non-Pregnant Adult - Juvenile	-0.026	-0.099	0.046	0.789

Model 8: aov (log FTM ~ Life Stage, data = 3 males, number of samples = 1230)

Variable	Sum Sq	df	F	*p*-value	η^2
Life Stage	3.94	1	113	<0.001	0.085
Residuals	42.7	1228			

Life Stage	Difference	Lower Limit	Upper Limit	*p*-value
Juvenile - Adult	-0.235	-0.279	-0.192	<0.001

Model 9: aov (log FTM ~ Month + Age, data = 3 males, number of samples = 1230)

Variable	Sum Sq	df	F	*p*-value	η^2
Month	3.86	11	13.5	<0.001	0.072
Age (year)	11.9	10	45.7	<0.001	0.255
Residuals	31.4	1208			

Month	Difference	Lower Limit	Upper Limit	*p*-value
2-1	-0.022	-0.101	0.057	0.999
3-1	0.030	-0.044	0.104	0.974
4-1	0.023	-0.051	0.097	0.997
5-1	0.059	-0.016	0.134	0.295
6-1	0.140	0.063	0.218	<0.001
7-1	0.073	-0.005	0.151	0.090
8-1	0.067	-0.010	0.143	0.161
9-1	0.036	-0.039	0.111	0.922
10-1	0.071	-0.006	0.149	0.104
11-1	-0.033	-0.109	0.044	0.964
12-1	-0.065	-0.142	0.013	0.218

3-2	0.052	-0.021	0.125	0.453
4-2	0.045	-0.028	0.119	0.676
5-2	0.081	0.006	0.156	0.020
6-2	0.162	0.085	0.239	<0.001
7-2	0.095	0.018	0.173	0.004
8-2	0.089	0.013	0.165	0.008
9-2	0.058	-0.017	0.133	0.319
10-2	0.094	0.016	0.171	0.004
11-2	-0.010	-0.086	0.065	1.000
12-2	-0.043	-0.120	0.035	0.817
4-3	-0.007	-0.075	0.061	1.000
5-3	0.029	-0.040	0.099	0.969
6-3	0.110	0.038	0.182	<0.001
7-3	0.043	-0.029	0.116	0.726
8-3	0.036	-0.034	0.107	0.874
9-3	0.006	-0.064	0.076	1.000
10-3	0.041	-0.030	0.113	0.767
11-3	-0.063	-0.133	0.008	0.139
12-3	-0.095	-0.167	-0.023	0.001
5-4	0.036	-0.034	0.106	0.879
6-4	0.117	0.044	0.189	<0.001
7-4	0.050	-0.023	0.123	0.522
8-4	0.043	-0.028	0.114	0.705
9-4	0.013	-0.057	0.083	1.000
10-4	0.048	-0.024	0.120	0.567
11-4	-0.056	-0.127	0.015	0.289
12-4	-0.088	-0.160	-0.016	0.004
6-5	0.081	0.007	0.155	0.017
7-5	0.014	-0.060	0.088	1.000
8-5	0.007	-0.065	0.080	1.000
9-5	-0.023	-0.095	0.048	0.996
10-5	0.012	-0.061	0.086	1.000
11-5	-0.092	-0.164	-0.019	0.002
12-5	-0.124	-0.197	-0.050	<0.001
7-6	-0.067	-0.144	0.010	0.157
8-6	-0.074	-0.149	0.001	0.059
9-6	-0.104	-0.178	-0.030	<0.001
10-6	-0.069	-0.145	0.007	0.122
11-6	-0.173	-0.247	-0.098	<0.001
12-6	-0.205	-0.281	-0.129	<0.001
8-7	-0.007	-0.082	0.069	1.000
9-7	-0.037	-0.112	0.037	0.893

10-7	-0.002	-0.078	0.075	1.000
11-7	-0.106	-0.181	-0.031	<0.001
12-7	-0.138	-0.214	-0.061	<0.001
9-8	-0.030	-0.103	0.042	0.968
10-8	0.005	-0.070	0.080	1.000
11-8	-0.099	-0.172	-0.026	<0.001
12-8	-0.131	-0.206	-0.056	<0.001
10-9	0.035	-0.038	0.109	0.918
11-9	-0.069	-0.141	0.004	0.083
12-9	-0.101	-0.174	-0.027	<0.001
11-10	-0.104	-0.179	-0.029	<0.001
12-10	-0.136	-0.212	-0.060	<0.001
12-11	-0.032	-0.107	0.043	0.963

Age (year)	Difference	Lower Limit	Upper Limit	*p*-value
1-0	-0.050	-0.169	0.070	0.962
2-0	0.373	0.259	0.486	<0.001
4-0	0.282	0.185	0.380	<0.001
5-0	0.263	0.168	0.358	<0.001
6-0	0.171	0.075	0.268	<0.001
7-0	0.161	0.064	0.259	<0.001
8-0	0.293	0.196	0.390	<0.001
9-0	0.158	0.061	0.256	<0.001
10-0	0.079	-0.023	0.180	0.313
11-0	0.407	0.221	0.594	<0.001
2-1	0.422	0.314	0.531	<0.001
4-1	0.332	0.241	0.424	<0.001
5-1	0.313	0.223	0.402	<0.001
6-1	0.221	0.131	0.311	<0.001
7-1	0.211	0.120	0.302	<0.001
8-1	0.343	0.252	0.434	<0.001
9-1	0.208	0.116	0.300	<0.001
10-1	0.128	0.032	0.224	<0.001
11-1	0.457	0.274	0.641	<0.001
4-2	-0.090	-0.174	-0.007	0.022
5-2	-0.110	-0.191	-0.029	<0.001
6-2	-0.201	-0.283	-0.119	<0.001
7-2	-0.211	-0.295	-0.128	<0.001
8-2	-0.080	-0.163	0.003	0.074
9-2	-0.214	-0.298	-0.130	<0.001
10-2	-0.294	-0.383	-0.205	<0.001

11-2	0.035	-0.145	0.214	1.000
5-4	-0.019	-0.076	0.037	0.990
6-4	-0.111	-0.169	-0.053	<0.001
7-4	-0.121	-0.180	-0.062	<0.001
8-4	0.011	-0.049	0.070	1.000
9-4	-0.124	-0.184	-0.064	<0.001
10-4	-0.204	-0.271	-0.137	<0.001
11-4	0.125	-0.045	0.295	0.384
6-5	-0.092	-0.145	-0.038	<0.001
7-5	-0.102	-0.157	-0.046	<0.001
8-5	0.030	-0.026	0.085	0.813
9-5	-0.105	-0.161	-0.048	<0.001
10-5	-0.184	-0.248	-0.121	<0.001
11-5	0.144	-0.024	0.313	0.173
7-6	-0.010	-0.067	0.047	1.000
8-6	0.122	0.065	0.178	<0.001
9-6	-0.013	-0.071	0.045	1.000
10-6	-0.093	-0.157	-0.028	<0.001
11-6	0.236	0.067	0.405	<0.001
8-7	0.132	0.073	0.190	<0.001
9-7	-0.003	-0.062	0.057	1.000
10-7	-0.083	-0.149	-0.017	0.003
11-7	0.246	0.077	0.416	<0.001
9-8	-0.135	-0.194	-0.075	<0.001
10-8	-0.214	-0.280	-0.148	<0.001
11-8	0.114	-0.055	0.284	0.522
10-9	-0.080	-0.147	-0.013	0.006
11-9	0.249	0.079	0.419	<0.001
11-10	0.329	0.157	0.501	<0.001

Model 10: aov (logFTM ~ Month + Age, data = 1 male, number of samples = 1102)

Variable	Sum Sq	df	F	*p*-value	η^2
Month	3.70	11	14.1	<0.001	0.119
Age (year)	5.54	7	33.1	<0.001	0.155
Residuals	25.9	1083			

Month	Difference	Lower Limit	Upper Limit	*p*-value
2-1	-0.038	-0.116	0.040	0.912
3-1	-0.002	-0.076	0.071	1.000

4-1	-0.013	-0.087	0.060	1.000
5-1	0.053	-0.021	0.126	0.442
6-1	0.143	0.067	0.218	<0.001
7-1	0.121	0.043	0.198	<0.001
8-1	0.093	0.018	0.168	0.003
9-1	0.077	0.002	0.152	0.041
10-1	0.107	0.030	0.184	<0.001
11-1	0.000	-0.075	0.076	1.000
12-1	-0.050	-0.126	0.025	0.571
3-2	0.036	-0.040	0.111	0.929
4-2	0.025	-0.051	0.100	0.996
5-2	0.091	0.015	0.167	0.005
6-2	0.181	0.103	0.259	<0.001
7-2	0.159	0.079	0.239	<0.001
8-2	0.131	0.054	0.209	<0.001
9-2	0.115	0.037	0.192	<0.001
10-2	0.145	0.066	0.224	<0.001
11-2	0.038	-0.039	0.116	0.902
12-2	-0.012	-0.090	0.066	1.000
4-3	-0.011	-0.082	0.060	1.000
5-3	0.055	-0.016	0.126	0.310
6-3	0.145	0.073	0.218	<0.001
7-3	0.123	0.048	0.198	<0.001
8-3	0.095	0.023	0.168	0.001
9-3	0.079	0.007	0.152	0.019
10-3	0.109	0.035	0.184	<0.001
11-3	0.003	-0.070	0.075	1.000
12-3	-0.048	-0.121	0.025	0.592
5-4	0.066	-0.005	0.137	0.094
6-4	0.156	0.083	0.229	<0.001
7-4	0.134	0.059	0.209	<0.001
8-4	0.106	0.034	0.179	<0.001
9-4	0.090	0.018	0.163	0.003
10-4	0.120	0.046	0.195	<0.001
11-4	0.014	-0.059	0.087	1.000
12-4	-0.037	-0.110	0.036	0.894
6-5	0.090	0.017	0.163	0.003
7-5	0.068	-0.007	0.143	0.122
8-5	0.040	-0.032	0.113	0.810
9-5	0.024	-0.049	0.097	0.995
10-5	0.054	-0.020	0.129	0.421
11-5	-0.052	-0.125	0.020	0.439

12-5	-0.103	-0.176	-0.030	<0.001
7-6	-0.022	-0.099	0.055	0.999
8-6	-0.050	-0.124	0.025	0.557
9-6	-0.066	-0.141	0.008	0.140
10-6	-0.036	-0.112	0.041	0.931
11-6	-0.142	-0.217	-0.068	<0.001
12-6	-0.193	-0.268	-0.118	<0.001
8-7	-0.028	-0.104	0.049	0.990
9-7	-0.044	-0.121	0.033	0.774
10-7	-0.014	-0.092	0.065	1.000
11-7	-0.120	-0.197	-0.043	<0.001
12-7	-0.171	-0.248	-0.094	<0.001
9-8	-0.016	-0.091	0.058	1.000
10-8	0.014	-0.062	0.090	1.000
11-8	-0.093	-0.167	-0.018	0.003
12-8	-0.143	-0.218	-0.068	<0.001
10-9	0.030	-0.046	0.107	0.979
11-9	-0.076	-0.151	-0.002	0.039
12-9	-0.127	-0.201	-0.052	<0.001
11-10	-0.107	-0.183	-0.030	<0.001
12-10	-0.157	-0.234	-0.080	<0.001
12-11	-0.050	-0.125	0.024	0.546

Age (year)	Difference	Lower Limit	Upper Limit	*p*-value
5-4	-0.012	-0.062	0.039	0.997
6-4	-0.102	-0.153	-0.050	<0.001
7-4	-0.108	-0.161	-0.054	<0.001
8-4	0.023	-0.031	0.076	0.903
9-4	-0.115	-0.170	-0.061	<0.001
10-4	-0.192	-0.253	-0.132	<0.001
11-4	0.164	0.011	0.318	0.026
6-5	-0.090	-0.138	-0.041	<0.001
7-5	-0.096	-0.146	-0.045	<0.001
8-5	0.034	-0.016	0.085	0.424
9-5	-0.104	-0.155	-0.053	<0.001
10-5	-0.181	-0.238	-0.123	<0.001
11-5	0.176	0.024	0.328	0.011
7-6	-0.006	-0.058	0.046	1.000
8-6	0.124	0.073	0.176	<0.001
9-6	-0.014	-0.066	0.038	0.993
10-6	-0.091	-0.149	-0.033	<0.001

11-6	0.266	0.113	0.418	<0.001
8-7	0.130	0.077	0.183	<0.001
9-7	-0.008	-0.062	0.046	1.000
10-7	-0.085	-0.145	-0.025	<0.001
11-7	0.272	0.118	0.425	<0.001
9-8	-0.138	-0.192	-0.085	<0.001
10-8	-0.215	-0.275	-0.155	<0.001
11-8	0.141	-0.012	0.295	0.095
10-9	-0.077	-0.137	-0.017	0.003
11-9	0.280	0.126	0.433	<0.001
11-10	0.357	0.201	0.512	<0.001

Model 11: aov (log FGM ~ Month + Age, data = 3 males, number of samples = 1230)

Variable	Sum Sq	df	F	*p*-value	η^2
Month	3.86	11	9.17	<0.001	0.048
Age (year)	32.5	10	85.0	<0.001	0.393
Residuals	46.2	1208			

Month	Difference	Lower Limit	Upper Limit	*p*-value
2-1	-0.006	-0.102	0.089	1.000
3-1	0.025	-0.065	0.114	0.999
4-1	0.133	0.043	0.222	<0.001
5-1	0.129	0.037	0.220	<0.001
6-1	0.157	0.063	0.252	<0.001
7-1	0.089	-0.006	0.183	0.093
8-1	0.053	-0.040	0.146	0.779
9-1	0.036	-0.055	0.128	0.979
10-1	0.025	-0.069	0.119	0.999
11-1	-0.006	-0.099	0.086	1.000
12-1	0.002	-0.092	0.096	1.000
3-2	0.031	-0.058	0.120	0.993
4-2	0.139	0.050	0.228	<0.001
5-2	0.135	0.044	0.226	<0.001
6-2	0.164	0.070	0.257	<0.001
7-2	0.095	0.001	0.189	0.047
8-2	0.059	-0.033	0.151	0.621
9-2	0.043	-0.048	0.134	0.930
10-2	0.031	-0.063	0.124	0.995
11-2	0.000	-0.092	0.092	1.000

12-2	0.009	-0.085	0.102	1.000
4-3	0.108	0.026	0.191	0.001
5-3	0.104	0.020	0.188	0.003
6-3	0.133	0.045	0.220	<0.001
7-3	0.064	-0.024	0.152	0.422
8-3	0.028	-0.058	0.114	0.996
9-3	0.012	-0.073	0.096	1.000
10-3	0.000	-0.087	0.087	1.000
11-3	-0.031	-0.116	0.055	0.990
12-3	-0.022	-0.110	0.065	1.000
5-4	-0.004	-0.089	0.081	1.000
6-4	0.025	-0.063	0.112	0.999
7-4	-0.044	-0.133	0.044	0.894
8-4	-0.080	-0.166	0.006	0.100
9-4	-0.096	-0.181	-0.011	0.011
10-4	-0.108	-0.196	-0.021	0.003
11-4	-0.139	-0.225	-0.053	<0.001
12-4	-0.130	-0.218	-0.043	<0.001
6-5	0.029	-0.061	0.118	0.996
7-5	-0.040	-0.130	0.050	0.951
8-5	-0.076	-0.164	0.012	0.171
9-5	-0.092	-0.179	-0.006	0.025
10-5	-0.104	-0.193	-0.015	0.008
11-5	-0.135	-0.222	-0.047	<0.001
12-5	-0.126	-0.216	-0.037	<0.001
7-6	-0.069	-0.162	0.024	0.388
8-6	-0.105	-0.195	-0.014	0.009
9-6	-0.121	-0.211	-0.031	<0.001
10-6	-0.133	-0.225	-0.041	<0.001
11-6	-0.164	-0.254	-0.073	<0.001
12-6	-0.155	-0.248	-0.063	<0.001
8-7	-0.036	-0.127	0.056	0.982
9-7	-0.052	-0.142	0.038	0.763
10-7	-0.064	-0.157	0.029	0.507
11-7	-0.095	-0.186	-0.004	0.033
12-7	-0.086	-0.179	0.007	0.099
9-8	-0.016	-0.104	0.072	1.000
10-8	-0.028	-0.119	0.062	0.997
11-8	-0.059	-0.148	0.030	0.571
12-8	-0.051	-0.141	0.040	0.805
10-9	-0.012	-0.101	0.078	1.000
11-9	-0.043	-0.130	0.045	0.913

12-9	-0.034	-0.124	0.056	0.985
11-10	-0.031	-0.121	0.060	0.994
12-10	-0.022	-0.115	0.070	1.000
12-11	0.008	-0.082	0.099	1.000

Age (year)	Difference	Lower Limit	Upper Limit	*p*-value
1-0	-0.065	-0.210	0.080	0.937
2-0	0.301	0.163	0.439	<0.001
4-0	0.106	-0.012	0.224	0.127
5-0	0.227	0.111	0.343	<0.001
6-0	0.223	0.107	0.340	<0.001
7-0	0.125	0.007	0.243	0.027
8-0	-0.096	-0.214	0.021	0.230
9-0	-0.145	-0.263	-0.026	0.004
10-0	-0.195	-0.318	-0.071	<0.001
11-0	-0.145	-0.371	0.081	0.599
2-1	0.366	0.234	0.497	<0.001
4-1	0.171	0.060	0.282	<0.001
5-1	0.292	0.184	0.400	<0.001
6-1	0.288	0.179	0.398	<0.001
7-1	0.190	0.079	0.301	<0.001
8-1	-0.031	-0.142	0.079	0.998
9-1	-0.080	-0.191	0.031	0.422
10-1	-0.130	-0.246	-0.013	0.015
11-1	-0.080	-0.303	0.142	0.986
4-2	-0.195	-0.296	-0.093	<0.001
5-2	-0.074	-0.172	0.025	0.355
6-2	-0.077	-0.177	0.022	0.298
7-2	-0.176	-0.277	-0.075	<0.001
8-2	-0.397	-0.498	-0.296	<0.001
9-2	-0.446	-0.547	-0.344	<0.001
10-2	-0.495	-0.603	-0.388	<0.001
11-2	-0.446	-0.664	-0.228	<0.001
5-4	0.121	0.053	0.189	<0.001
6-4	0.117	0.048	0.187	<0.001
7-4	0.019	-0.053	0.091	0.999
8-4	-0.202	-0.274	-0.131	<0.001
9-4	-0.251	-0.324	-0.178	<0.001
10-4	-0.301	-0.382	-0.220	<0.001
11-4	-0.251	-0.457	-0.045	0.004
6-5	-0.004	-0.069	0.061	1.000

7-5	-0.102	-0.170	-0.035	<0.001
8-5	-0.323	-0.391	-0.256	<0.001
9-5	-0.372	-0.440	-0.304	<0.001
10-5	-0.422	-0.499	-0.345	<0.001
11-5	-0.372	-0.577	-0.168	<0.001
7-6	-0.098	-0.167	-0.029	<0.001
8-6	-0.320	-0.389	-0.251	<0.001
9-6	-0.368	-0.438	-0.298	<0.001
10-6	-0.418	-0.496	-0.340	<0.001
11-6	-0.369	-0.574	-0.164	<0.001
8-7	-0.221	-0.293	-0.150	<0.001
9-7	-0.270	-0.342	-0.198	<0.001
10-7	-0.320	-0.400	-0.239	<0.001
11-7	-0.270	-0.476	-0.065	0.001
9-8	-0.048	-0.120	0.024	0.526
10-8	-0.098	-0.178	-0.018	0.004
11-8	-0.049	-0.254	0.157	1.000
10-9	-0.050	-0.131	0.031	0.662
11-9	0.000	-0.206	0.206	1.000
11-10	0.049	-0.160	0.258	1.000

Model 12: aov (logFGM ~ Month + Age, data = 1 male, number of samples = 1102)

Variable	Sum Sq	df	F	*p*-value	η^2
Month	2.00	11	6.44	<0.001	0.034
Age (year)	28.2	7	143	<0.001	0.463
Residuals	30.5	1083			

Month	Difference	Lower Limit	Upper Limit	*p*-value
2-1	0.019	-0.066	0.104	1.000
3-1	0.025	-0.055	0.104	0.997
4-1	0.028	-0.052	0.107	0.993
5-1	0.090	0.011	0.170	0.012
6-1	0.151	0.069	0.233	<0.001
7-1	0.112	0.027	0.196	<0.001
8-1	0.064	-0.017	0.146	0.291
9-1	0.048	-0.034	0.129	0.747
10-1	0.033	-0.051	0.116	0.982
11-1	0.014	-0.068	0.096	1.000
12-1	0.018	-0.064	0.100	1.000

3-2	0.005	-0.077	0.088	1.000
4-2	0.008	-0.074	0.091	1.000
5-2	0.071	-0.011	0.154	0.169
6-2	0.132	0.048	0.216	<0.001
7-2	0.092	0.006	0.179	0.025
8-2	0.045	-0.039	0.129	0.842
9-2	0.029	-0.056	0.113	0.994
10-2	0.013	-0.073	0.100	1.000
11-2	-0.005	-0.090	0.079	1.000
12-2	-0.001	-0.086	0.083	1.000
4-3	0.003	-0.074	0.080	1.000
5-3	0.066	-0.011	0.143	0.179
6-3	0.127	0.048	0.205	<0.001
7-3	0.087	0.006	0.168	0.024
8-3	0.040	-0.039	0.118	0.888
9-3	0.023	-0.055	0.102	0.998
10-3	0.008	-0.073	0.089	1.000
11-3	-0.011	-0.089	0.068	1.000
12-3	-0.007	-0.086	0.073	1.000
5-4	0.063	-0.014	0.140	0.244
6-4	0.123	0.044	0.203	<0.001
7-4	0.084	0.002	0.165	0.037
8-4	0.037	-0.042	0.115	0.935
9-4	0.020	-0.059	0.099	1.000
10-4	0.005	-0.076	0.086	1.000
11-4	-0.014	-0.093	0.065	1.000
12-4	-0.010	-0.089	0.070	1.000
6-5	0.061	-0.018	0.140	0.331
7-5	0.021	-0.060	0.103	0.999
8-5	-0.026	-0.105	0.053	0.995
9-5	-0.043	-0.121	0.036	0.834
10-5	-0.058	-0.139	0.023	0.451
11-5	-0.076	-0.155	0.003	0.070
12-5	-0.072	-0.152	0.007	0.113
7-6	-0.040	-0.123	0.044	0.926
8-6	-0.087	-0.168	-0.006	0.023
9-6	-0.103	-0.184	-0.022	0.002
10-6	-0.119	-0.202	-0.036	<0.001
11-6	-0.137	-0.218	-0.056	<0.001
12-6	-0.133	-0.214	-0.052	<0.001
8-7	-0.047	-0.131	0.036	0.784
9-7	-0.064	-0.147	0.020	0.336

10-7	-0.079	-0.164	0.006	0.101
11-7	-0.098	-0.181	-0.014	0.008
12-7	-0.094	-0.177	-0.010	0.014
9-8	-0.017	-0.097	0.064	1.000
10-8	-0.032	-0.115	0.051	0.984
11-8	-0.050	-0.131	0.031	0.669
12-8	-0.046	-0.127	0.035	0.779
10-9	-0.015	-0.098	0.068	1.000
11-9	-0.034	-0.115	0.047	0.969
12-9	-0.030	-0.111	0.051	0.989
11-10	-0.019	-0.102	0.064	1.000
12-10	-0.015	-0.098	0.069	1.000
12-11	0.004	-0.077	0.085	1.000

Age (year)	Difference	Lower Limit	Upper Limit	*p*-value
5-4	0.116	0.061	0.171	<0.001
6-4	0.115	0.058	0.171	<0.001
7-4	0.018	-0.040	0.076	0.982
8-4	-0.207	-0.265	-0.149	<0.001
9-4	-0.252	-0.311	-0.194	<0.001
10-4	-0.300	-0.365	-0.234	<0.001
11-4	-0.224	-0.390	-0.057	0.001
6-5	-0.001	-0.054	0.052	1.000
7-5	-0.098	-0.153	-0.043	<0.001
8-5	-0.323	-0.377	-0.268	<0.001
9-5	-0.368	-0.424	-0.313	<0.001
10-5	-0.416	-0.478	-0.353	<0.001
11-5	-0.340	-0.505	-0.174	<0.001
7-6	-0.097	-0.153	-0.041	<0.001
8-6	-0.322	-0.377	-0.266	<0.001
9-6	-0.367	-0.424	-0.311	<0.001
10-6	-0.415	-0.478	-0.351	<0.001
11-6	-0.339	-0.504	-0.173	<0.001
8-7	-0.225	-0.282	-0.167	<0.001
9-7	-0.270	-0.329	-0.212	<0.001
10-7	-0.318	-0.383	-0.253	<0.001
11-7	-0.242	-0.408	-0.075	<0.001
9-8	-0.046	-0.104	0.013	0.252
10-8	-0.093	-0.158	-0.028	<0.001
11-8	-0.017	-0.183	0.149	1.000
10-9	-0.047	-0.113	0.018	0.360

11-9	0.029	-0.138	0.195	1.000
11-10	0.076	-0.093	0.245	0.874
